# Fruit weight is controlled by *Cell Size Regulator* encoding a novel protein that is expressed in maturing tomato fruits

**DOI:** 10.1371/journal.pgen.1006930

**Published:** 2017-08-17

**Authors:** Qi Mu, Zejun Huang, Manohar Chakrabarti, Eudald Illa-Berenguer, Xiaoxi Liu, Yanping Wang, Alexis Ramos, Esther van der Knaap

**Affiliations:** 1 Department of Horticulture and Crop Science, The Ohio State University, Wooster Ohio, United States of America; 2 Institute of Plant Breeding, Genetics and Genomics, University of Georgia, Athens Georgia, United States of America; 3 Department of Horticulture, University of Georgia, Athens Georgia, United States of America; The University of North Carolina at Chapel Hill, UNITED STATES

## Abstract

Increases in fruit weight of cultivated vegetables and fruits accompanied the domestication of these crops. Here we report on the positional cloning of a quantitative trait locus (QTL) controlling fruit weight in tomato. The derived allele of *Cell Size Regulator* (*CSR-*D) increases fruit weight predominantly through enlargement of the pericarp areas. The expanded pericarp tissues result from increased mesocarp cell size and not from increased number of cell layers. The effect of *CSR* on fruit weight and cell size is found across different genetic backgrounds implying a consistent impact of the locus on the trait. In fruits, *CSR* expression is undetectable early in development from floral meristems to the rapid cell proliferation stage after anthesis. Expression is low but detectable in growing fruit tissues and in or around vascular bundles coinciding with the cell enlargement stage of the fruit maturation process. *CSR* encodes an uncharacterized protein whose clade has expanded in the Solanaceae family. The mutant allele is predicted to encode a shorter protein due to a 1.4 kb deletion resulting in a 194 amino-acid truncation. Co-expression analyses and GO term enrichment analyses suggest association of CSR with cell differentiation in fruit tissues and vascular bundles. The derived allele arose in *Solanum lycopersicum* var *cerasiforme* and appears completely fixed in many cultivated tomato’s market classes. This finding suggests that the selection of this allele was critical to the full domestication of tomato from its intermediate ancestors.

## Author summary

Starting about 10,000 years ago, during the Neolithic period, human societies began the transformation from a hunting and gathering-dependent lifestyle to an agrarian lifestyle. This transformation was accompanied by plant and animal domestication. Tomato shows a huge increase in fruit weight that has arisen as a consequence of its domestication. We identified a gene that encodes a poorly characterized protein that controls fruit weight in tomato. The mutation that led to the increase in fruit weight arose early during the cultivation of tomato and is now incorporated in all large tomato varieties. The gene regulates cell size in the fruit and is called *Cell Size Regulator*. The increases in cell size are proposed to relate to cellular maturation that accompanies fruit growth.

## Introduction

Rapid morphological diversification among closely related organisms often arise in response to strong selection pressures such as those imposed by domestication. Starting approximately 10,000 years ago, during the Neolithic period, human societies began the transformation from a hunting and gathering-dependent lifestyle to an agrarian lifestyle which was accompanied by plant and animal domestication [1, 2]. The process of domestication is associated with the taming of wild relatives and the selections of types that benefit human use in terms of food production and clothing as well as shelter and companionship. Specifically for vegetable and fruit crops, the domesticated plants feature much larger produce, reduced plant branching and often a determinate growth habit [1].

The fully wild ancestor of tomato (*Solanum pimpinellifolium*) is indigenous largely to the coastal regions of Ecuador and Peru [3, 4]. Selections to the intermediate type, *S*. *lycopersicum* var *cerasiforme*, took place in the Andean mountain region of Ecuador and Northern Peru and further selections to the earliest domesticate type, *S*. *lycopersicum* var *lycopersicum*, took place in Central America [4]. Continued selections after the initial domestication events led to a huge diversity of size and shape with fruit weight increasing as much as a 1,000 fold [5].

Even though the fruit is a terminal structure, the parameters that determine its final shape and weight are rooted throughout the plant’s lifespan. The development of the fruit starts as early as the formation of the inflorescence and floral meristem 3 weeks after sowing [6, 7]. In tomato, floral development from inflorescence meristem to anthesis-stage flowers takes place in approximately 3 weeks whereas fruit development from opened flower to ripe fruit lasts between 4.5 and 7 weeks depending on the genotype [7, 8]. Final fruit morphology is the result of a combination of processes that regulate meristem organization, overall cell division rates and duration, cell shape and cell expansion. Meristem organization processes that lead to larger meristems support the formation of a bulkier fruit. For example, natural mutations in the tomato orthologs of the meristem architecture genes *WUSCHEL* (*WUS*) and *CLAVATA3* (*CLV3*) lead to fruit with more locules associated with large and flat fruits [9]. Induced mutations in genes in the same WUS-CLV3 pathway also support the notion that enlarging meristems lead to bulkier fruit [10]. Distinct from meristem organization are the cell division, cell shape and cell expansion processes which can be controlled at multiple time points throughout the ontogeny of the ovary and fruit. These cellular processes are typically defined to take place before or after anthesis [11]. Whereas details of cell division, cell shape and cell expansion are not well described for tomato ovary development, processes following pollination and fertilization of the ovules are much better understood. The initial stages of fruit development following successful pollination and fertilization are marked by a rapid increase in cell proliferation through reinitiating cell divisions [7, 8]. In most fruit tissues such as the pericarp, cell division ceases 5 to 10 days after anthesis with the exception of the epidermal cell layer. Following this rapid cell division stage, fruit growth continues by extensive cell enlargements that last for three to five weeks until the fruit ripening stage [7, 8]. Thus far, two natural mutations in tomato impact cell number by increasing the number of cell layers in the pericarp of the fruit. This increase in cell layers is achieved by changing cell division rates and/or duration in the developing ovary or fruit [12, 13]. CNR/FW2.2 changes cell layers in the ovary walls and these organs are already larger at anthesis [13]. SlKLUH/FW3.2 also impacts cell layers mainly in the pericarp but of the developing fruit instead of the ovary [12]. In addition to fruit size, the mutation in *SlKLUH* is associated with delayed ripening suggesting that the duration of cell division is extended [12]. Derived alleles for *CNR/FW2*.*2* and *SlKLUH/FW3*.*2* are resulting in expression level changes as gene expression is different in the NILs. Moreover, association mapping led to the most significant polymorphisms in the promoter of these two fruit weight genes [12, 14]. Thus far, natural mutations in tomato genes that impact cell size have not yet been identified. However, increases in cell size are associated with tomato domestication and the emergence of larger fruit [15].

The increase in size from a 1 to 2-mm wide ovary to a 5 to 10-cm wide fruit is predominantly the result of the dramatic increase in cell enlargement in the pericarp that follows the cell proliferation stage [7, 8]. In the developing fruit, the transition and maintenance of cell enlargement is not well understood. On the other hand, the transition from proliferation to enlargement is better described in root development [16, 17]. Cells differentiate from the root meristem zone to the transition zone which is controlled by the antagonistic action of auxin and cytokinin [16, 18]. Certain cytokinin signaling regulators, the B-type Arabidopsis Response Regulators (ARRs), appear to play crucial roles in synchronizing the entry into the endoreduplication cycle in roots of Arabidopsis [16, 17, 19]. In roots, the transition zone also marks the initiation of endoreduplication, a process marked by DNA replication without nuclear division. This process is known to occur in maturing cells and results in higher nuclear ploidy levels by bypassing chromatid segregation and cytokinesis [20, 21]. Endoreduplication is proposed to occur in many multicellular organisms as a potential mechanism for cell enlargement and cellular differentiation. In particular for tomato, the proper development of the tomato fruit is intimately associated with endoreduplication [15, 22].

We here report on the cloning and characterization of a novel tomato fruit weight gene *Cell Size Regulator* (*CSR*). This quantitative trait locus had been identified in a prior study as a minor QTL explaining only 8% of the phenotypic variance [23]. Despite its small effect on the trait in a segregating F_2_ population, the heritability in progeny studies was high, facilitating the fine mapping and eventual cloning of the gene. The derived allele *CSR*-D increases fruit mass through cell enlargement which is accompanied by small increases in nuclear ploidy level of pericarp cells. Expression and co-expression analyses with *CSR* suggests a role in cell differentiation during the later stages of fruit development, including vascular development. We propose that CSR impacts cellular differentiation leading to a potentially indirect effect on endoreduplication during tomato fruit maturation. The *CSR*-D mutation likely arose in *S*. *lycopersicum* var *cerasiforme* and became entirely fixed in the large fruited tomato germplasm suggesting that this improvement was an important step towards the domestication of tomato from the cherry type to the cultivated *S*. *lycopersicum* var *lycopersicum*.

## Results

### *Solyc11g071940* underlies *fw11*.*3* and controls weight by increasing cell size

**Fig 1:**
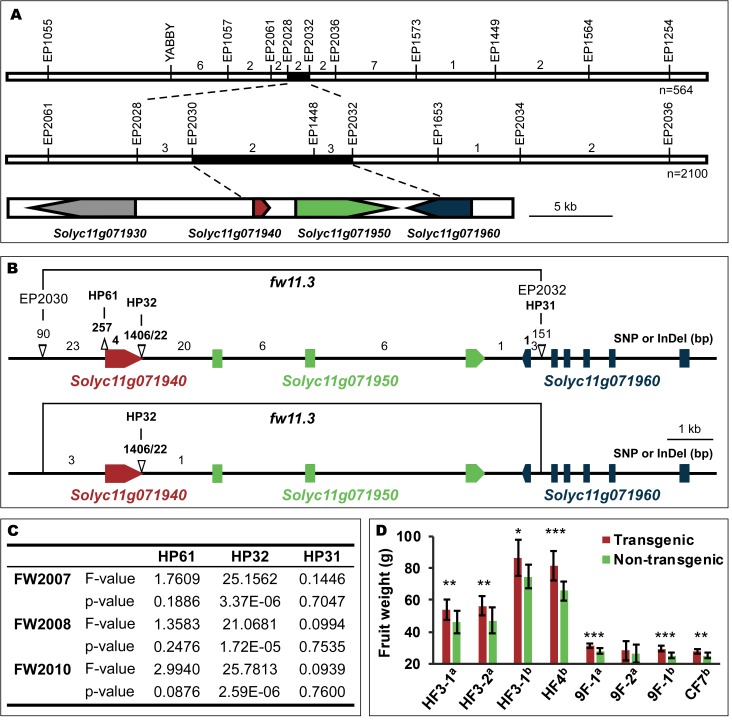
Cloning of the *fw11*.*3* QTL. (A) The *fw11*.*3* locus was mapped to the EP2028-EP2032 interval on tomato chromosome 11. The *fw11*.*3* locus was further narrowed down to EP2030-EP2032 interval including two and a half open reading frames. Numbers above the chromosome indicate number of recombinant plants in the respective interval that were progeny tested. The black region delineates the fine mapped locus on the genome. The three candidate genes are shown in red, green and blue. (B) The DNA polymorphisms at the *fw11*.*3* locus among LA1589 and Howard German/Rio Grande (upper) or Yellow Pear and Gold Ball Livingston (lower) segregating populations. Bold numbers above the chromosome indicate polymorphisms in the coding region; non-bold numbers indicate polymorphisms in the non-coding region. EP2030, EP2030, HP61, HP32 and HP31 denote markers. Δ: deletion or insertion. (C) Association mapping results of fruit weight with markers HP61, HP32 and HP31. The years used to collect the data are shown as FW2007, FW2008 and FW2010. (D) Transgenic complementation tests. Average fruit weight of 10 to 13 plants from T_1_ generation transgenic and non-transgenic sib plants cultivated under the same growing conditions are shown. Error bar: standard deviation. HF, Howard German background and 9F/CF, VIR347 background. Data collected during the 2013 field season is denoted with an “a”. Data collected during the 2014 field season is denoted with a “b”. Significance determined by paired *t*-tests and transgenic (transgenically carrying the *CSR*-D allele) plants were compared to their non-transgenic (*CSR*-WT) sibs. *, p ≤ 0.05; **, p ≤ 0.01; ***, p ≤ 0.001.

The *fw11*.*3* locus was previously mapped to a 149 kb region on chromosome 11 [24] and further fine-mapped to a 13kb region between markers EP2030 and EP2032 (Fig 1A, S1 Table). This region included two full open reading frames, *Solyc11g071940* and *Solyc11g071950*, and the partial *Solyc11g071960*. The common nucleotide polymorphisms among the *fw11*.*3* segregating populations were a large deletion at the 3’ end of *Solyc11g071940* and four SNPs in the non-coding region surrounding the same gene (Fig 1B; S1 Table). We next conducted an association study to determine whether the partial gene deletion was correlated with fruit weight variation in the tomato germplasm. Using a population that was developed to conduct association studies for fruit weight in tomato [25], the 3’ deletion of *Solyc11g071940* was shown to be significantly associated with fruit enlargement and over three years of fruit weight evaluations (Fig 1C). The association of the large deletion in *Solyc11g071940* with fruit weight implies that this gene underlies *fw11*.*3*.

Detailed sequence analyses showed that the derived allele carried a 1,406 bp deletion and 22 bp insertion resulting in a 194 amino-acid truncation of the predicted wild type protein. Interestingly, the truncation might not result in a loss-of-function of *fw11*.*3*, as the derived allele was partially dominant over the wild type allele [24]. To further corroborate that *Solyc11g071940* was the likely candidate *FW11*.*3* gene and that the derived allele constituted a gain-of-function mutation, we constructed transgenic complementation lines by transferring the partially dominant allele of *Solyc11g071940* to the lines in the wild type *fw11*.*3* background. Fifteen independent transgenic complementation lines (T_0_) were obtained of which four progeny families (T_1_) were evaluated at least once. Fruit weight was significantly increased in nearly all transgenic progeny lines compared to their non-transgenic sibs (Fig 1D, S2 Table). Therefore, the results from the transgenic complementation lines demonstrate that *Solyc11g071940* controls tomato fruit size and that the deletion found in the derived allele is indeed acting in a dominant manner. This finding supports the notion that *fw11*.*3*-D functions as a gain-of-function mutation.

**Fig 2:**
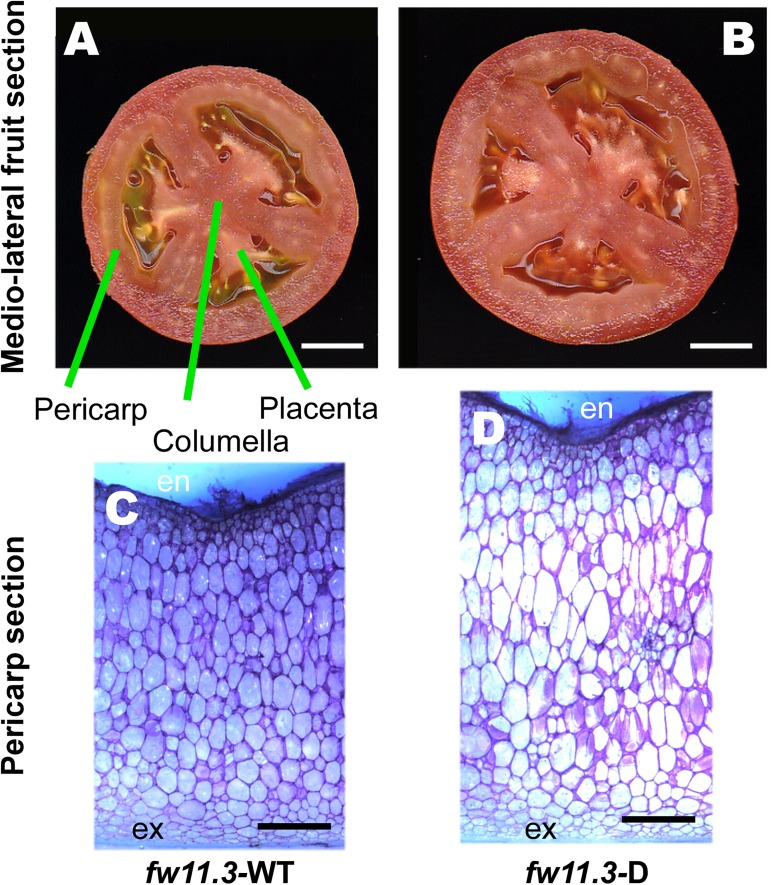
Fruit and cellular structure of *fw11*.*3* NILs in the Howard German background. (A) Medio-lateral section of *fw11*.*3*-WT fruit. Size bar = 1 cm. The pericarp, columella and placenta tissues are shown. (B) Medio-lateral section of *fw11*.*3*-D fruit. Size bar = 1 cm. (C) Hand cut section of a *fw11*.*3*-WT pericarp from a representative mature green fruit stained with toluidine blue. Size bar = 1 mm. (D) Hand cut section of a *fw11*.*3*-D pericarp from a representative mature green fruit stained in toluidine blue. Size bar = 1 mm. The inner epidermis is denoted as “en” whereas the outer epidermis is denoted as “ex”. The mesocarp are represented by the cells between the inner and outer epidermis.

As expected, the fruit weight, fruit area and fruit perimeter were significantly larger in the *fw11*.*3*-D nearly isogenic lines (NIL) in the cultivated background (Table 1). To evaluate the effect of the QTL in the wild species background, we developed NILs where *fw11*.*3*-D was introgressed in the mostly LA1589 background, an accession of *S*. *pimpinellifolium* that is the closest fully wild ancestor species of cultivated tomato. These NILs were either differing for just *fw11*.*3* or were fixed for the derived alleles at *fw2*.*2* and *fw3*.*2* while differing at the *fw11*.*3* locus. Similar to the NILs in the cultivated background, fruit weight, fruit area and fruit perimeter were significantly larger in the *fw11*.*3*-D in the wild species background (Table 1). To evaluate which parts of the fruit were enlarged by the derived allele, we analyzed the pericarp, columella and placenta areas in the NILs (Fig 2). The pericarp area was significantly enlarged in the NILs that carry the derived allele in both cultivated and wild tomato species backgrounds. By contrast, the columella and placenta area were not consistently different in the NILs (Table 1). Because the effect of *fw11*.*3* was most pronounced in the pericarp in all the NILs, we evaluated whether increased cell layers (number) in the mesocarp or increased cell size contributed to the larger pericarp area values. Our data showed that the number of cell layers was mostly unaltered in NILs in both cultivated and wild species backgrounds whereas mesocarp cell size was increased in all the lines carrying the derived allele of *fw11*.*3* (Fig 2, Table 2). Moreover, transgenic lines that complemented the large fruit phenotype also showed increased cell sizes compared to non-transgenic control. Thus, the role of *FW11*.*3* is to increase cell size and therefore we named *Solyc11g071940* to *Cell Size Regulator (CSR)*.

**Table 1:** Fruit structure analyses in medio-lateral sections of the *fw11*.*3* NILs.

Population (background)	12S114 (RG)		12S116 (RG)		12S147 (HG)		12S148 (HG)		15S107 (LA1589)			
Allele	*fw11*.*3*-D	*fw11*.*3*-WT	*fw11*.*3*-D	*fw11*.*3*-WT	*fw11*.*3*-D	*fw11*.*3*-WT	*fw11*.*3*-D	*fw11*.*3*-WT	*fw2*.*2/fw3*.*2/fw11*.*3*-D	*fw2*.*2/fw3*.*2/fw11*.*3*-WT	*fw11*.*3*-D	*fw11*.*3*-WT
Trait												
Fruit Weight (g)	88.0 ± 5.3	73.2 ± 1.9***	66.3 ± 3.8	53.0 ± 2.6****	91.6 ± 4.5	72.6 ± 2.2****	88.4 ± 2.2	68.2 ± 4.6****	2.13 ± 0.13	1.49 ± 0.10****	1.51 ± 0.12	1.26 ± 0.04**
Fruit Perimeter (cm)	17.7 ± 0.64	16.6 ± 0.3**	16.0 ± 0.3	14.8 ± 0.5***	15.8 ± 0.4	14.3 ± 0.3****	16.0 ± 0.4	14.3 ± 0.4****	5.11 ± 0.22	4.64 ± 0.10**	4.61 ± 0.17	4.31 ± 0.1*
Fruit Area (cm^2^)	22.2 ± 1.5	19.6 ± 0.7**	18.2 ± 0.7	15.7 ± 1***	17.9 ± 1	14.7 ± 0.5****	18.4 ± 0.9	14.7 ± 0.9****	1.89 ± 0.16	1.56 ± 0.07**	1.55 ± 0.11	1.34 ± 0.06*
Pericarp Area (cm^2^)	11.08 ± 0.92	9.53 ± 0.35**	9.89 ± 0.30	8.16 ± 0.47****	8.57 ± 0.42	6.87 ± 0.29****	8.15 ± 0.36	6.59 ± 0.51****	0.82 ± 0.08	0.70 ± 0.03*	0.62 ± 0.05	0.55 ± 0.02*
Pericarp Area/Total Area	0.50 ± 0.02	0.49 ± 0.01	0.54 ± 0.02	0.52 ± 0.02	0.48 ± 0.02	0.47 ± 0.01	0.44 ± 0.02	0.45 ± 0.03	0.43 ± 0.01	0.45 ± 0.003*^a^	0.40 ± 0.01	0.40 ± 0.004
Pericarp Thickness (cm)	0.77 ± 0.05	0.72 ± 0.02*	0.78 ± 0.03	0.69 ± 0.03***	0.67 ± 0.03	0.58 ± 0.02****	0.61 ± 0.03	0.54 ± 0.02***	0.21 ± 0.01	0.20 ± 0.003	0.27 ± 0.01	0.25 ± 0.005*
Placenta and Columella Area (cm^2^)	3.52 ± 0.28	3.00 ± 0.22**	2.26 ± 0.25	2.19 ± 0.29	2.32 ± 0.25	1.96 ± 0.20*	2.91 ± 0.4	2.30 ± 0.3**	0.065 ± 0.008^b^	0.050 ± 0.009*^,^^b^	0.033 ± 0.005^b^	0.033 ± 0.002^b^
Placenta and Columella Area/Total Area	0.16 ± 0.01	0.15 ± 0.01	0.12 ± 0.01	0.14 ± 0.01*^,^^a^	0.13 ± 0.01	0.13 ± 0.01	0.16 ± 0.02	0.16 ± 0.01	0.034 ± 0.002^b^	0.032 ± 0.005^b^	0.021 ± 0.003^b^	0.025 ± 0.002^b^

RG, Rio Grande

HG, Howard German

LA1589, an accession of the wild species *S*. *pimpinellifolium*. Significance determined by paired *t*-Test and compared to *fw11*.*3*-D in the same NIL.

*, p ≤ 0.05

**, p ≤ 0.01

***, p ≤ 0.001

****, p ≤ 0.0001.

^a^ Note the value is larger for the *fw11*.*3*-WT.

^b^ Values reflect the columella area only.

**Table 2:** Cell size and cell number comparisons in the pericarp of *fw11*.*3* NILs and VIR347 transgenic lines.

** **	**12S147 Howard German NILs**			**14S81 Rio Grande NILs**			
	***fw11*.*3*-D**	***fw11*.*3*-WT**	***P-*value**	***fw11*.*3*-D**		***fw11*.*3*-WT**	***P*-value**
	**n = 5**	**n = 5**	** **	**n = 11**		**n = 9**	** **
Cell layers	18.8 ± 0.6	18.6 ± 0.6	0.664	30.39 ± 2.12		29.62 ± 1.34	0.360
Largest cell size (mm^2^)	0.21 ± 0.02	0.16 ± 0.02	0.006	0.16 ± 0.02		0.14 ± 0.01	0.048
Average cell size (mm^2^)	0.10 ± 0.02	0.08 ± 0.01	0.042	0.07 ± 0.012		0.057 ± 0.009	0.015
	**15S107 LA1589 NILs**						
	***fw2*.*2/fw3*.*2/fw11*.*3*-D**	***fw2*.*2/fw3*.*2/fw11*.*3*-WT**	***P-*value**	***fw11*.*3*-D**		***fw11*.*3*-WT**	***P*-value**
	**n = 5**	**n = 6**	** **	**n = 5**		**n = 6**	** **
Cell layers	16.34 ± 0.50	15.82 ± 0.72	0.237	13.86 ± 0.70		13.02 ± 0.18	0.030
Largest cell size (mm^2^)	0.054 ± 0.006	0.045 ± 0.005	0.034	0.050 ± 0.005		0.035 ± 0.004	6.66E-04
Average cell size (mm^2^)	0.031 ± 0.007	0.025 ± 0.003	0.118	0.027 ± 0.003		0.016 ± 0.001	0.001
** **	**14S68 VIR347 transformed lines**			**14S70 VIR347 transformed lines**			
	**Transgenic**	**Non-transgenic**	***P*-value**	**Transgenic**	**Non-transgenic**		***P*-value**
	**n = 10**	**n = 10**	** **	**n = 8**	**n = 9**		** **
Cell layers	18.69 ± 0.78	18.63 ± 0.73	0.855	19.67 ± 0.31	19.51 ± 0.72		0.560
Largest cell size (mm^2^)	0.12 ± 0.02	0.09 ± 0.01	0.002	0.12 ± 0.01	0.09 ± 0.01		5.55E-05
Average cell size (mm^2^)	0.069 ± 0.014	0.052 ± 0.011	0.007	0.067 ± 0.009	0.046 ± 0.007		8.42E-05

Cell layers reflect the mesocarp cell number in the pericarp between the epidermis and the endodermis.

Largest cell size is based on the average size of the six largest cells in the pericarp found in the hand cut sections.

Average cell size reflects the average cell size in the mesocarp by dividing the number of cells by a set area. The set area was 6 mm^2^ for 12S147; 9.466 mm^2^ for 14S81; 0.543 mm^2^ and 0.506 mm^2^ for the LA1589 NILs respectively; and 2.036 mm^2^ for 14S68 and 14S70.

"n" reflects the number of plants evaluated. For each plant, at least four sections from three fruits were evaluated.

#### Other phenotypes associated with *CSR* in the *fw11*.*3* NILs

To determine whether *fw11*.*3*-D affected other plant growth characteristics, we evaluated several traits such as yield per plant and leaf size. In addition to fruit weight, *fw11*.*3* controlled fruit number per plant such that *fw11*.*3*-WT produced more fruit than *fw11*.*3*-D (S3 Table). Overall yield per plant was not altered, which implied that increased fruit weight was compensated by fewer fruit per plant. Higher fruit number could have resulted from increased length of the side shoots which in turn would have led to more inflorescences even though the latter trait was not significantly different in the NILs. It was unlikely that *fw11*.*3* controlled the source-sink relationship because reducing the number of fruit concommittantly led to larger fruits in both *fw11*.*3*-WT and *fw11*.*3*-D backgrounds (S3 Table). The effect on fruit weight was manifested late in development, namely after anthesis since ovary size was the same in the *fw11*.*3* NILs, and without an effect on ripening time. Other architectural and morphological traits were not consistently altered in the NILs implying that the effect of *fw11*.*3* was nearly exclusively on fruit weight by increasing cell size (S3 Table).

Cells in fruits of many plant species undergo endoreduplication leading to increased nuclear ploidy levels. The increase in ploidy and nuclear size is positively correlated to cell size in tomato [15]. To determine whether *CSR* might control cell size in the pericarp by increasing the nuclear size, we determined the changes in ploidy levels in mature green fruit collected from the *fw11*.*3* NIL plants. At the mature green stage, the fruit had reached its largest size which occurred between 35 (mature green) and 40 to 43 DPA (ripening) in the Rio Grande background [26]. The flow cytometry results showed the most prevalent ploidy levels of 8C, 16C, and 32C but also some nuclei with levels as high as 512C in the tomato pericarp (P10 in S1 Fig). Over multiple experiments, the Rio Grande *fw11*.*3*-D NIL pericarps featured more nuclei at higher ploidy level (64C to 512C) compared to *fw11*.*3*-WT for at least one C-value (S4 Table). In one replicate, the increased number of higher ploidy level nuclei was accompanied by a reduced number of lower ploidy nuclei in *fw11*.*3*-D compared to *fw11*.*3*-WT. The endoreduplication index (EI') in two out of five replicates was significantly increased, suggesting that CSR-D leads to more nuclei at higher ploidy levels compared to WT. We also evaluated changes in nuclei ploidy levels in the pericarp of the NILs in the LA1589 wild species background. The EI' was significantly higher in one of two sets of NILs, which is consistent with the results from the Rio Grande NILs. Nuclear ploidy levels were also evaluated in columella tissues (S4 Table). In the Rio Grande background, nuclei ploidy levels at certain higher C-values were the same or decreased in *fw11*.*3*-D, contrary to the ploidy level observations in the pericarp. In all, EI' in columella was never significantly increased in *fw11*.*3*-D in either cultivated or wild species background. Combined, these results implied that the effect of *fw11*.*3* on endoreduplication might be indirect and if so, it only affects the cells in the tomato pericarp.

### Protein sequence and phylogeny analysis of CSR

**Fig 3:**
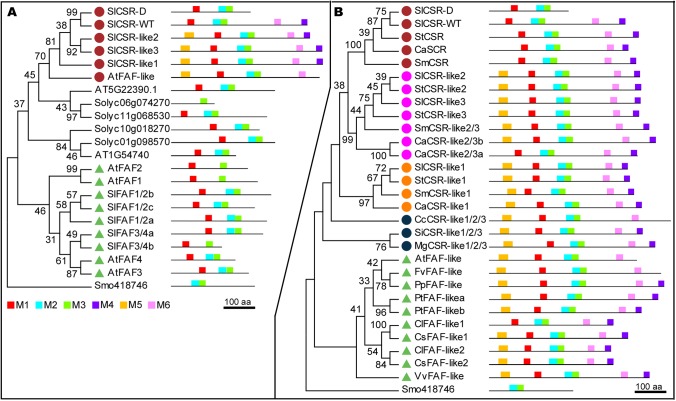
Phylogenic and motif analysis of FAF domain-containing proteins. (A) Phylogenic tree of tomato and Arabidopsis proteins, using FAF domain sequences in the tree construction. Motif structure is based on full length protein sequences. (B) Phylogenic tree and motif analysis of CSR-like proteins found in different plant species: *Solanum lycopersicum* (Sl), *Solanum tuberosum* (St), *Solanum melongena* (Sm), *Capsicum annuum* (Ca), *Coffea canephora* (Cc), *Sesamum indicum* (Si), *Mimulus guttatus* (Mg), *Arabidopsis thaliana* (At), *Fragaria vesca* (Fv), *Prunus persica* (Pp), *Populus trichocarpa* (Pt), *Citrullus lanatus* (Cl), *Cucumis sativus* (Cs), *Vitis vinifera* (Vv), and *Selaginella moellendorffii* (Smo). *Selaginella moellendorfii* (Smo) is used as outgroup. The same colored dots or triangles represent same subclade. Scale bar: 100 amino acids. Renamed proteins: Solyc06g073940 (SlCSR-like1), Solyc01g009260 (SlCSR-like2), Solyc01g009270 (SlCSR-like3), Solyc06g084280 (SlFAF1/2a), Solyc06g008990 (SlFAF1/2b), Solyc09g065140 (SlFAF1/2c), Solyc01g079740 (SlFAF3/4a), Solyc06g054310 (SlFAF3/4b), PGSC0003DMP400005394 (StCSR), CA11g16000 (CaCSR), Sme2.5_00683.1_g00009 (SmCSR), PGSC0003DMP400023251 (StCSR-like2), PGSC0003DMP400023246 (StCSR-like3), Sme2.5_01340.1_g00001 (SmCSR-like2/3), CA01g13730 (CaCSR-like2/3b), CA01g13720 (CaCSR-like2/3a), PGSC0003DMP400010456 (StCSR-like1), Sme2.5_00076.1_g00022 (SmCSR-like1), CA06g22610 (CaCSR-like1), Cc01g07830 (CcCSR-like1/2/3), Sin1010620 (SiCSR-like1/2/3), mgv1a004589m (MgCSR-like1/2/3), mrna23163.1-v1.0-hybrid (FvFAF-like), ppa002898m (PpFAF-like), Potri.009G016600 (PtFAF-like_a), Potri.001G216000.2 (PtFAF-like_b), Cla008617 (ClFAF-like1), Csa6M426380 (CsFAF-like1), Cla007326 (ClFAF-like2), Csa1M635920 (CsFAF-like2), VIT206s0009g003101 (VvFAF-like).

The predicted CSR protein featured a domain that is recognized as the FAF domain (formely known as DUF3049, Pfam accession PF11250). The tomato genome harbored 13 predicted proteins that represented FAF domain family. In Arabidopsis, the FAF domain was found in 10 proteins which included four FANTASTIC FOUR (FAF) and a single FAF-like protein. In tomato, three genes were recognized as *CSR* paralogs because their proteins were closely related to one another and shared similar motif patterns. We named these three paralogs *CSR-like1*, *CSR-like2* and *CSR-like3* (Fig 3). At the protein level, the MEME motifs 2 and 3 represented the FAF domain whereas motif 6 defined the CSR family proteins. Motifs 4 and 5 were also often found in members of the CSR clade. The single copy Arabidopsis FAF-like protein was the most similar to the tomato CSR clade, whereas the four FAFs formed a separate clade (Fig 3A). Likely orthologs of the four Arabidopsis FAFs were identified in tomato, including three *SlFAF1/2* and two *SlFAF3/4*. The FAF proteins have been recognized as controlling shoot meristem size [27].

Only one *CSR* member was found in Arabidopsis whereas four *CSR* paralogs were found in tomato. To further explore the apparent expansion of the CSR family, we searched databases for protein members that were closely related to CSR and included motifs 4 and/or 6. The most likely orthologs of tomato *CSR* and *CSR-like 1* to *3* were found in other Solanaceous species namely potato, eggplant and chile pepper (Fig 3B). When comparing species with sequenced genomes outside the Solanaceae family but within the Asterids clade, only one copy of CSR was found in coffee (Gentianales), sesame and mimulus (Lamiales) (Fig 3B). However, based on motif structure, this single paralog appeared more similar to the *CSR-like1* to *3* encoded proteins than to CSR. For species in the Rosids clade, most of them carried only one copy of a *CSR-like* except watermelon, cucumber and poplar. These results suggested a single common ancestor of *CSR* that evolved independently in Rosids and Asterids clades, and only expanded to four in the Solanaceace family.

### Expression analysis of *CSR* and related genes

**Fig 4:**
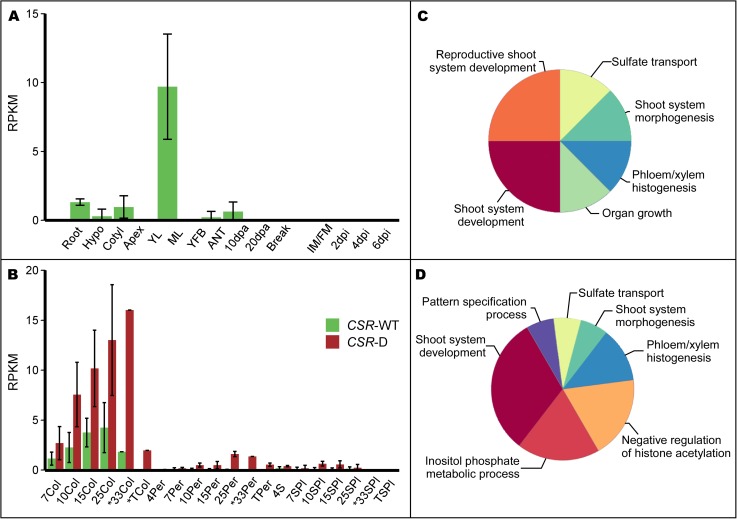
Expression of *CSR* in tomato plant organs and fruit tissues. (A) Expression of *CSR-*WT in tomato LA1589 plant tissues. Root: root; Hypo: hypocotyl; Cotyl: cotyledon; Apex: shoot apex including the SAM and youngest leaves; YL: young leaf; ML: mature leaf; YFB: young floral buds from 10 days after initiation and younger; ANT: whole flower at anthesis; 10 and 20 dpa: 10 and 20 days post anthesis developing fruit, respectively; Break: breaker stage fruit which is before turning color; IM/FM: inflorescence and floral meristem; 2, 4, and 6 dpi: 2, 4, and 6 days post initiation flower buds, respectively. Error bar indicates standard deviation. (B) CSR transcript accumulation in *fw11*.*3* NILs fruit tissues during fruit development. Error bar: standard deviation. *The 33Col, 33Per, 33SPl and TCol samples represent one replicate. Col: columella; Per: pericarp; S: seeds; SPl: seeds and placenta; T: turning stage fruit. Numbers associated with the sample names represent the fruit developmental stage as days post anthesis. Hence, 7Col represents columella tissue collected 7 days post anthesis. (C) ClueGO enrichement in the *CSR-D* coexpression cluster. (D) ClueGO enrichment in the *CSR-WT* coexpression cluster. The dimension of the pie chart wedges is proportional to the number of terms included in each category. The most significant term of the group was used for annotation.

To further understand the role in tomato fruit growth, we evaluated the temporal and spatial expression patterns of *CSR*. Despite its effect only on fruit weight, *CSR*-WT expression was not detected in whole developing fruit (Fig 4A). Low expression however, was detected in mature leaf tissues (Fig 4A, S5 Table). Moreover, analyses of other RNA-seq datasets showed that *CSR* expression was undetectable in tomato meristems (http://tomatolab.cshl.edu/efp/cgi-bin/efpWeb.cgi), as well as ovary and 4 days old fruit tissues collected by laser captured microscopy (http://ted.bti.cornell.edu/cgi-bin/TFGD/digital/experiment.cgi?ID=D009). These results demonstrated that *CSR* expression was undetectable from meristems, young floral buds, anthesis to the cell proliferation stage of fruit development. Although *CSR* expression was undetectable when evaluating the whole fruit, we expected gene expression in certain tissues within this organ at later developmental stages. Indeed when sampling the pericarp, columella and seed&placenta tissues separately from maturing fruits, the expression of *CSR* was found to be low but detectable. *CSR* was expressed the highest in the columella where its expression increased between 7 to 33 days post anthesis (DPA), and decreased again at the turning stage (Fig 4B). *CSR* was also expressed in pericarp and seed/placenta tissues albeit at low or very low levels (Fig 4B). Interestingly, *CSR*-D showed higher expression compared to *CSR*-WT but with a similar profile in both columella and pericarp. Recently, higher resolution expression information in developing tomato fruits (from J.K.C Rose, Cornell University) also supported the notion that *CSR*-D was highest expressed in the columella. Moreover, the expression was particularly high in the vascular bundles of the developing tomato pericarp (S2 Fig).

Of all the genes that encode the FAF motif, *CSR-like1* was the highest expressed in nearly all plant tissues examined including seedling tissues (cotyledons, hypocotyl, shoot apex and roots) and in the columella of developing fruit (S5 Table). In developing fruit, *CSR-like1* featured the most similar expression dynamic as *CSR*, even though *CSR-like1* was often 15 fold or higher expressed than *CSR*-D (S5 Table). Expression of *CSR-like2* and *CSR-like3* was specifically detected in hypocotyl, cotyledon and shoot apex (Fig 4A, S5 Table). In all, *CSR* was expressed dynamically in developing fruit and *CSR*-D allele was higher expressed than *CSR*-WT allele. On the other hand, tomato *FAF1/2a* showed the highest expression in floral meristem tissues and early developing floral buds; *FAF1/2c* was particularly well expressed in emerging leaves; and *FAF3/4a* was well expressed in seedling tissues as well as floral meristem and early floral buds. In sum, *CSR* and *CSR-like* genes show distinct expression in maturing tissues whereas the *FAFs* are highest expressed in meristems and young tisues.

#### Co-expression patterns of *CSR* in developing fruit

DESeq2 and other differential gene expression methods showed few genes that were significantly differentially expressed at different time points and tissue types throughout fruit development when contrasting the *fw11*.*3-D* and *fw11*.*3-WT* NILs [26]. And very few of those were consistent among more than one time point or tissue type. Instead, we sought to identify genes that were expressed with similar dynamics as *CSR*. Genes with common expression patterns could share related functions as they may control or be controlled by similar processes in the plant [28]. To identify potential shared developmental and/or metabolic processes with *CSR*, we conducted co-expression cluster analyses and then performed functional enrichment using the RNA-Seq data from the *fw11*.*3-D* and *fw11*.*3-WT* NILs separately. Cluster analyses showed that *CSR*-D had a 99.3% probability to be part of cluster 11 which was comprised of 493 genes (probability of 90% or higher) (S3A Fig, S1 Dataset). Functional enrichment using ClueGO identified six pathways that were significantly enriched. In the *CSR*-D co-regulated cluster, the pathway “shoot system development” was especially highly enriched with 31 genes, 17 of which encoded putative transcription factors (Fig 4C, S2 Dataset). The enriched pathway “phloem or xylem histogenesis” was also of interest due to the relatively high expression of *CSR*-D in the vascular tissues (S2 Fig). In this pathway, six genes of which four encoded putative transcription factors were found.

We next identified the *CSR*-WT co-expressed genes using the RNA-seq data set that was generated with the *fw11*.*3*-WT NIL. Because many of the genes were found in both the *CSR*-D and the *CSR*-WT co-regulated clusters, we expected similar pathways to be enriched. Of the 558 *CSR*-WT cluster genes with a probability of 90% or higher, 63% (352) were shared with the *CSR*-D co-expressed cluster 11 (S1 Dataset, S3B Fig). Indeed, the pathway “shoot system development” was similarly enriched in the *CSR*-D and *CSR*-WT clusters. Likewise, the pathway “phloem or xylem histogenesis” was enriched in both clusters (Fig 4D). These findings suggest a role for CSR in regulating cell size during shoot and vascular development in growing tissues.

Genes of interest in the co-regulated clusters that were not part of an enriched GO term were also found. One gene of particular interest was *CSR-like1*, which showed similar expression patterns in developing fruits as *CSR* (S5 Table). Also, several putative orthologs in the cytokinin signaling and biosynthesis pathway such as *LOG3* (Solyc11g069570), *IPT5* (Solyc01g080150), *WOL* (Solyc04g008110), *ARR12* (Solyc07g005140) and *ARR1* or *ARR2* (Solyc01g0655540) were found in one or both *CSR* co-expressed gene clusters. Moreover, a likely ortholog of an auxin efflux carrier *PILS5* (Solyc03g032080), an auxin signaling gene *ARF11* (Solyc05g0560400), and a gene encoding a pleckstrin domain-containing protein (Solyc08g066860) which is thought to be a component of the auxin canalization pathway were found in the CSR co-regulated gene clusters.

### Origin of *CSR*-D during the evolution of tomato

**Fig 5:**
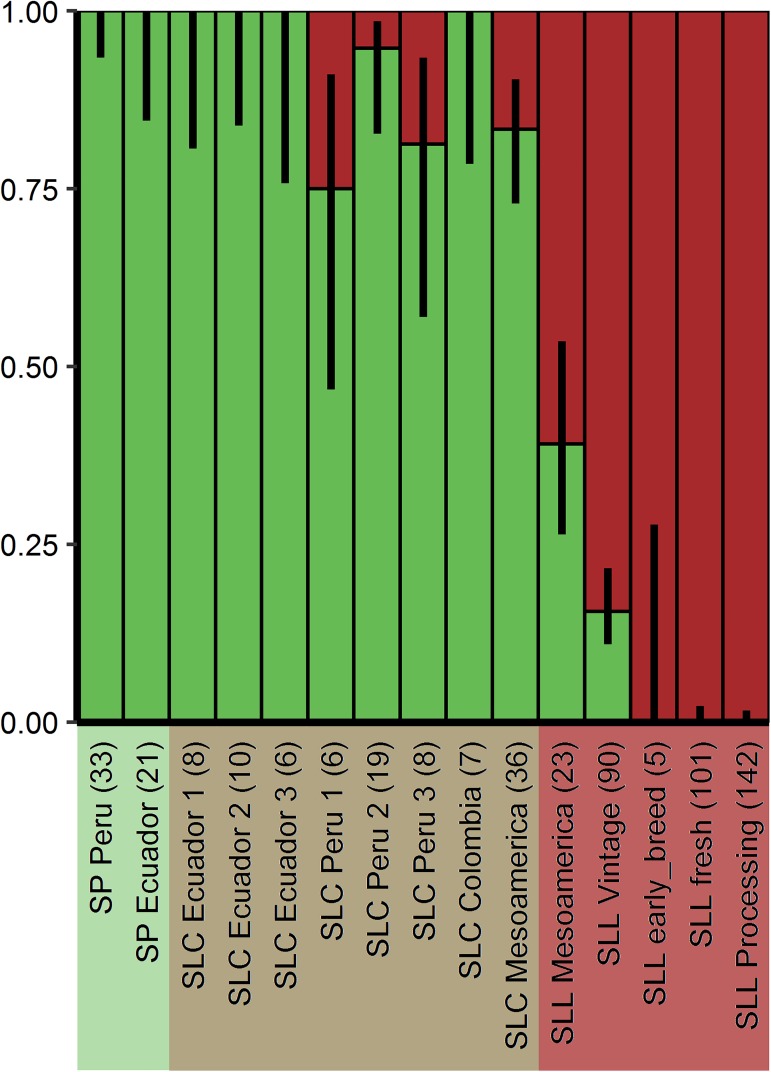
Distribution of *CSR* wild type and derived allele in the tomato germplasm. Ancestral allele in green, derived allele in burgundy. Black lines show binomial confidence intervals at 95%. Background colors highlight different species: *S*. *pimpinellifolium* (light green), *S*. *lycopersicum* var. *cerasiforme* (light brown) and *S*. *lycopersicum* var. *lycopersicum* (pink). Number of accessions in each category is given in parenthesis above the different genetically distinct classes [4].

Fruit weight was an important selection criterion that drove the evolution of tomato from a wild relative bearing small fruit to the large tomatoes found in grocery stores today. To determine whether selections for the derived allele of *CSR* might have taken place, we evaluated the origin and distribution of *CSR*-D in wild, semi-domesticated, early landraces as well as modern breeding germplasm (Fig 5). The mutation was found in low frequency in *S*. *lycopersicum* var *cerasiforme* from Peru and Mesoamerica. However, the distribution of the derived allele frequency greatly increased in the Mesoamerican domesticated landraces and is now completely fixed in the large fruited contemporary germplasm. The contemporary germplasm included processing and fresh market tomatoes, the latter which were comprised of globe/large used for slicing and plum tomatoes used for soups and stews [4]. These results imply that *CSR*-D arose late but became rapidly fixed during the selections by early farmers.

## Discussion

In this study, we demonstrated by fine mapping, association mapping and plant transformation experiments that the tomato *fw11*.*3* QTL is controlled by *Solyc11g071940* encoding a largely uncharacterized protein. The 1.4 kb deletion caused a 194 amino acid truncation at the C-terminus of the encoded protein creating a partial dominant allele. The heavier fruit was predominantly caused by increased pericarp area resulting from larger cells in this tissue, hence the name CSR which is short for Cell Size Regulator. Increases in cell size in the pericarp were correlated to increases in nuclear ploidy levels in this same tissue albeit that CSR’s effect on this process appears to be small. Fixation of the derived allele in cultivated tomato supported the notion that increased cell size mediated by CSR was indeed critical for the recent evolution of the crop.

### *CSR* coincides with the cell enlargement stage in developing tomato fruit

In roots, cellular differentiation occurs when cells progress from the meristematic (dividing) zone through the transition zone into the elongation and differentiation zone. Development of the tomato pericarp may follow a similar trajectory as described for the root: immediately following pollination and fertilization and after a short period of cell division, pericarp cells transition to the cell expansion stage. In small fruited tomatoes, the cell layers in the walls of the tomato increase from 10 to 20 in less than five days following pollination [7]. This period is followed by approximately 3.5 weeks of cell enlargement prior to ripening. Expression of *CSR* coincides with the cell expansion stage and therefore, this gene might be associated with cellular differentiation. A conserved domain FAF with unknown function has been identified in CSR and CSR-like proteins. This domain is named after the Arabidopsis FANTASTIC FOUR (FAF) proteins regulating shoot meristem size [27]. Whereas FAFs are expressed strongly in meristematic cells and young growing tissues, *CSR* expression was undetectable in these tissues and instead only found in maturing plant organs such as the fruits prior to ripening (S5 Table). Thus, the function of CSR and FAFs is not likely the same at the tissue level even though biochemically they might be involved in similar processes within the cell. Our research efforts focused on the effect of *CSR* in the pericarp because this tissue contributes greatly to overall fruit weight, and the area was significantly expanded through cell size increases in the *fw11*.*3*-D NILs. However, expression of *CSR* was the highest in the columella and not the pericarp (Fig 4). Recently, we obtained higher resolution expression of *CSR*-D in developing tomato fruit which showed that expression of this gene is high in and around vascular bundles in the pericarp. Thus, the high expression of *CSR* in the columella could be due to the relatively high vasculature density in columella tissues. We attempted to evaluate cell size in the columella which ranged from very small (in the center) to very large (towards the periphery). However, coumella structure varied a lot from fruit to fruit and therefore, it was not feasible to evaluate cell size similarly in the columella sections taken from different fruits. Since the entire vascular bundle was captured by laser captioned microdissection, it is not known where in or around the bundles *CSR* is expressed. To further understand the function of *CSR*, future studies should be directed to evaluate its role in vascular development and in the columella.

### CSR might be involved in the antagonistic action of auxin and cytokinin to regulate cell differentiation

Even though *CSR* encodes a protein of unknown function, co-expression analyses may have revealed the cellular processes that provide insights into the function of CSR. The antagonistic roles of auxin and cytokinin might possibly be associated with CSR function since several genes associated with these pathways were found in the co-expression clusters. For example in Arabidopsis root development, the B-type response regulators ARR2 and ARR12 play important roles in the mitotic exit by upregulating the expression of *CCS52A1*, an activator of the APC/C complex, and *SHY2/IAA3*, leading to inhibition of auxin signaling [17, 19]. Putative orthologs of *ARR2* (Solyc01g0655540) and *ARR12* (Solyc07g005140) were found in the *CSR* co-expressed dataset. Additional cytokinin signaling and biosynthetic proteins were identified including LOG3 (Solyc11g069570) of unknown function but thought to play a role in the biosynthesis of cytokinins, a cytokinin synthase protein IPT5 (Solyc01g080150) [29], a cytokinin receptor CRE/AHK4 (Solyc04g008110) [30], and a cytokinin sensitivity protein PRL1 (Solyc01g094480) [31] were found in the *CSR* gene clusters in the *fw11*.*3* NILs. Auxin signaling and response genes were found in these clusters as well, namely genes encoding auxin transport protein PILS5 (Solyc03g032080) and ABCB15 (Solyc02g087410) [32, 33], an auxin signaling protein ARF11 (Solyc05g0560400), a pleckstrin domain-containing protein (Solyc08g066860) that is involved in vascular patterning and auxin canalization [34], a HB-2 transcription factor (Solyc08g078300) involved in cell expansion and response to auxin [35, 36], IBR5 (Solyc12g005990) a dual specificity phosphatase that promotes auxin responses and acts as a regulator of organ size in Arabidopsis [37], and a KNOTTED-like protein 1 (Solyc04g077210) whose expression is repressed by auxin resulting in the promotion of leaf fate [38, 39]. GO term enrichment for genes in the *CSR* expression clusters also implied that plant vascular development is one of the processes that may be affected by *CSR* and *CSR-like1* which was consistent with its expression in this tissue. Indeed, deeper searches in the entire list of co-regulated genes led to the identification of additional genes that may be critical in vascular development. These include genes that encode putative orthologs of receptor-like kinases XIP1 (Solyc04g077010) involved in the maintenance of cell files or cell morphology in conductive elements [40] and BRI1-like 2 (Solyc04g008430) associated with the development of provascular/procambium cells [41–43], as well as several phloem expressed lectin genes (Solyc02g069020, Solyc02g069030, Solyc02g069060, Solyc02g031740). In addition, putative orthologs of genes involved in vascular development [44] were found in the dataset including *APL* (Solyc12g017370), *CNA* (Solyc12g044410) and *BRX-like 4* (Solyc12g044410). With *CSR* expression particularly high in vascular bundles and the columella (which is enriched for xylem and phloem tissues), these co-regulated genes suggest that *CSR* and perhaps *CSR-like1* play a role in cellular maturation in the vascular bundle leading indirectly to increases in pericarp cell size and nuclear ploidy. Therefore, *CSR* might be involved in the antagonistic effects of auxin and cytokinin as a mechanism for cellular differentiation and enlargement in different tissue types.

Cell enlargement is also associated with enhanced endoreduplication and larger fruit weights [15]. Ubiquitously found in both higher plants and animals, the function of endoreduplication is not well understood other than its association with increased cell size. The transition from cell proliferation to enlargement in roots coincides with the initiation of endoreduplication [16, 17, 19, 20]. Other studies have suggested a role for endoreduplication in enhanced metabolism [45] or to sustain growth under adverse conditions including pathogen attack [46, 47]. Even though the function is not well understood, the mechanisms regulating the core entry and progression of endoreduplication has been reasonably well established. Distinct stages of cell division are regulated by CYCLINS (CYC), Cyclin-Dependent Kinases (CDK) and CDK inhibitors (CKI). The onset and progression of endoreduplication is mediated by these same core cell cycle proteins through transcription factors regulating gene expression as well as regulators that control the ubiquitination of the proteins which then targets them for proteolytic degradation. Specifically, CYCLINS and CDKs that regulate the M stage are suppressed when endoreduplication is promoted [20, 48]. This suppression is mediated by activation of a specific E3 ubiquitin ligase named the Anaphase Promoting Complex/Cyclosome (APC/C) that leads to ubiquitination of the mitotic Arabidopsis proteins CDKB1;1 and CYCA2;3 which is then followed by proteasome-mediated protein degradation [49]. Activators of APC/C are for example *Cell Cycle Switch52 A1* (*CCS52A1*) encoding a WD-repeat protein and mutations or transcriptional downregulation leads to termination of cell expansion and reduced endoreduplication. It is unlikely, however that CSR plays an important role in endoreduplication because none of the known aforementioned endoreduplication genes such as those encoding CYCLINS, CDK and CKI or any related to APC/C proteasome complex and most of their transcriptional and translational regulators, were present in the *CSR* gene expression clusters (S1 Dataset) [26]. Moreover, the impact of *CSR*-D on enhancing endoreduplication was very small.

### Evolution of CSR in dicot plants

How did the *CSR* clade including the *FAF-like* genes evolve in plants? In our study, we observed that *CSR* expanded in the Solanaceae family resulting in four paralogs: *CSR-like1*, *CSR-like2*, *CSR-like3* and *CSR*. Among them, *CSR-like2* and *CSR-like3* might have been the result of a recent tandem duplication event because they shared high sequence similarity to one another and were located next to each other on chromosome 1. When compared with selected species in the Solanaceae family, most of the four paralogs corresponded to an ortholog in pepper, eggplant and potato. The clustered *CSR-like2* and *3* shared two orthologs in potato and pepper, but only one in eggplant. The eggplant genome sequence is not yet available and thus, it was possible that eggplant also carried two *CSR-like2/3* gene copies. As for other species in the Asterids clade, coffee, sesame and mimulus carried only one paralog of the *CSR* family genes. This result suggest that the duplication resulting in *CSR*, *CSR-like1* and *CSR-like2/3* occurred after the Solanales diverged from the Gentianales and Lamiales orders, but before the Solanaceae family split to tomato, potato, pepper and eggplant. A single *FAF-like* gene is found in monocot and dicot species and is proposed to be the ancestor to *FAF* [27] as well as the *CSR* clade based on our findings (Fig 3A). In fact, the predicted protein motif structure of FAF-like in Arabidopsis is more similar to CSR than to FAF. As *CSR* and *CSR-like* genes only expanded in the Solanaceae family, they may have evolved specific functions that are specific to the family. Regardless and as mentioned above, the CSR subclade of the FAF proteins may be involved in cellular differentiation and enlargement resulting from the antagonistic action of auxin and cytokinin. And this might be the unifying role of CSR/CSR-like/FAF-like genes in land plants.

## Materials & methods

### Plant materials

Tomato seeds or DNA were obtained from the TGRC (http://tgrc.ucdavis.edu/; *S*. *pimpinellifolium* LA1589); Tomato Growers Supply Co (Howard German, Rio Grande, Yellow Pear); Dr. Mathilde Causse (Institut National de la Recherche Agronomique-Avignon, France; Tomato core collection for association mapping [25]); Drs. Maria José Díez and Jose Blanca (Universitat Politècnica de València, Spain; Instituto de Conservación y Mejora de la Agrodiversidad Valenciana collection [4]); Dr. David Francis (The Ohio State University, United States; SolCAP collection [50]). Plants were grown in field (for fruit weight and plant evaluations) and greenhouse (for population development and evaluation of additional plant phenotypes) at Ohio Agricultural Research and Development Center (Wooster, OH USA) or at the University of Georgia (Athens, GA USA) under standard conditions. For the greenhouse, the plants were grown in 2-gallon pots using 15-9-12 Osmocote slow release supplemented by 20-20-20 fertilization in Fafard 3B growing media with supplemental lighting.

### Population development, fine mapping and progeny testing

To fine map *fw11*.*3*, a population derived from a cross between *S*. *lycopersicum* c.v. Howard German (HG) and the wild species *S*. *pimpinellifolium* accession LA1589 was used [51] (S4 Fig). New markers were developed for the fine mapping and were based on the tomato genome sequence and known marker sequences (S6 Table). Eight recombinants were obtained through screening of 1906 seedlings, and two additional recombinants were obtained through screening 732 seedlings. Fruit weight was compared between *fw11*.*3*-WT and *fw11*.*3*-D plants within each recombinant family using Student’s *t*-test (S1 Table). Additional mapping was conducted in populations derived from crosses between *S*. *lycopersicum* c.v. Rio Grande (RG) × LA1589 and Gold Ball Livingston (GBL) × Yellow Pear (YP). In RG, three recombinants were progeny tested (S1B Table) whereas another three recombinants were progeny tested from an GBL × YP F_2_ population [52] (S1C Table).

NILs in the cultivated background were developed from the fine mapping populations in the HG and RG populations and only differed at the locus of interest while all other parts of the genome were fixed. Resulting from initial backcrosses to the cultivated parent, 75% and 87.5% of the loci in these NILs were estimated to be fixed for the HG or RG parent, respectively. The HG NILs originated from BC_1_F_7_ 11S62-2 plant (S4 Fig), with the introgression region of around 36kb. The RG NILs originated from BC_2_F_5_ 12S114-5 plant in RG mapping population (S4B Fig), with the introgression region around 532kb. A second set of RG NILs were derived from the BC_2_F_6_ heterozygous plant 12S255-11, with the introgressed region of around 131kb.

The *fw11*.*3* NILs in LA1589 background were developed by marker-assisted selection after several generations of backcrossing and selfing. Breeding scheme for NIL development is schematically shown in S4C Fig. The entire introgressed region at *fw11*.*3* locus (73 kb) contained 11 annotated genes (ITAG2.4 tomato genome annotation release), including *CSR*. In all cases, the NILs were grown in a randomized plot design and therefore cultivated under the same conditions.

### Association mapping

The accessions used for the association mapping were a Core Collection that included 93 *S*. *pimpinellifolium*, *S*. *lycopersicum* var *cerasiforme* and *S*. *lycopersicum* var *lycopersicum* accessions [25] (S7 Table). Association mapping was performed using three InDel markers, HP61, HP32 and HP31 (S6 Table) found in the 13 kb region spanning the locus in a population described by [25]. Association analysis was performed using MLM model of TASSEL2.1 software [53]. Population structure matrix Q and kinship matrix K were generated with STRUCTURE 2.2 [54] and SPAGeDi [55], respectively. Twenty EST-SSR markers distributed throughout the genome were used to generate Q and K matrix [25].

### Plant transformation

Fosmid SL_FOS0119H09 (provided by Dr. J.J. Giovannoni, USDA-ARS, Ithaca NY) from tomato cultivar Heinz1706 spanned the *fw11*.*3*-D locus. To release the insert for transformation, the clone was digested with *Avr*II and *Sgr*AI corresponding to the *Solyc11g071940* coding region, 7,101-bp upstream region and 3,466-bp downstream region. The resulting 11,314 bp insert was cloned into the *Agrobacterium* transformation vector pHaoN, modified from pCAMBIA1300 (by adding the following selectable marker: P_nos_-KAN-T_nos_), linearized by digestion with *Xba*I and *Xma*I. The enzymers *Avr*II and *Xba*I, *Sgr*AI and *Xma*I are two pairs of isocaudomers that generate compatible ends after digestion. The complementation construct pH*ORF2* was transformed into VIR347, carrying the wild type allele of *fw11*.*3* similar to LA1589; and also in the HG NIL containing the LA1589 wild type allele (11S167 carrying *fw11*.*3*-WT, S4A Fig). Transformation was conducted at the Plant Transformation Core Research Facility at the University of Nebraska-Lincoln (Dr. T. Clemente) and independent transgenic lines were identified after Southern blot hybridizations of *Eco*RI and *Eco*RV-digested genomic DNA following standard procedures. For each transgenic family, 10 to 13 transgenic complementation plants and non-transgenic sib plants were identified from the T_1_ generation using marker assisted selection, transplanted in a random plot design, and evaluated under the same conditions. A total of four independent transgenic events (HF3, HF4, 9F, CF7) were evaluated in 2013 and 2014, and HF3 and 9F were replicated in both years. For the T_1_ transgenic lines from HF3-1 and HF4 grown in 2014, only a few fruit had matured at the time of harvest and 8 to 12 fruit per plant were weighted individually. Due to large variations among plants within same genotype, plants that carried the largest and smallest fruit of the same genotype were removed prior to statistical evaluations [26] (S2 Table). Average fruit weight per plant was calculated by divding the fruit number by total weight.

### Cytological evaluations of the *fw11*.*3* NILs and VIR347 transgenic complementation plants

Cell layer and mesocarp cell area were measured in pericarp of breaker-stage fruit of the *fw11*.*3* NILs, and the VIR347 transgenic and non-transgenic sib plants. Two to three slices per fruit and two representative fruit per plant were evaluated. Transverse sections of the pericarp (approximately 1 mm thick and 1 cm long) cut from the equatorial region were stained by adding a drop of 0.5% Toluidine Blue in 0.1% Na_2_CO_3_ solution for 1 to 2 seconds. The samples were rinsed with water to prevent staining of the internal cell layers. The stained sections were photographed using the attached digital camera (SPOT RT KE, Diagnostic Instruments) on the Leica MZFLIII dissecting microscope (Leica Microsystems, Switzerland). The cell layers were counted four times in each section from the exocarp to endocarp avoiding the vascular bundles. Largest cell size was measured by tracing the six largests cells with ImageJ. The average cell size was measured by counting the cell number in an equal sized rectangle and divided by the rectangular area. Student *t*-test were used to compare the cell number and cell size difference between the two genotypes.

### Ploidy analysis

Representative mature green fruit were used for ploidy analyses. Five slices of fresh pericarp tissue (1 mm thick slice of 0.5–1 cm^2^ area, avoiding septum tissue) from each fruit were chopped finely under 1.2 ml nuclei extraction buffer (100 mM Tris-Cl pH 7, 85 mM NaCl, 5 mM MgCl_2_, and 0.1% Triton X100), with a razor blade A 100 μm nylon mesh filter (Sysmex Partec GmbH, Görlitz, Germany) was used to filter the nuclei from the chopped tissue suspension (600 μL). Three μL of DAPI solution (0.2 mg/mL) was added to each sample prior to loading onto the BD LSRII flow cytometer (Biomedical Research Tower Facility, Columbus, OH) or the CyAn ADP flow cytometer (Beckman Coulter, Cytometry Shared Resource Laboratory, Athens GA) for fluorescence-activated cell sorting (FACS) analysis. 10,000 nuclei were counted using 405 nm laser excitation and blue emission filter 450/50, Only 3,000 nuclei were counted if gating became clogged (possibly due to debris interference). A 7 DPA fruit and mature leaves were used as internal control to calibrate nuclei content C-values for the RG NILs and LA1589 NILs, respectively. After manually adjusting the gating to exclude background noise, the histograms of different nuclei level (C-values) events were generated (S1 Fig). The percentage of each ploidy level from all nuclei counts was calculated. The lower C value nuclei were not evaluated as those peaks were not discernable above background noise. The percentage of each ploidy level was compared between *fw11*.*3*-D and *fw11*.*3*-WT. To calculate the Endoreduplication Index (EI), we modified the established formula by removing 2C and 4C ploidy levels and calculated EI as EI’ = [4C*1+ 8C*2+ 16C*3+ 32C*4+ 64C*5+ 128C*6+ 256C*7+ 512C*8] / [total counts from 4C to 512C] [56].

### Ovary, fruit, seed, plant architecture, leaf structure, and source-sink relationship analyses

Ovaries at anthesis were collected for size measurement. The middle part of ovaries were infltrated and fixed with 3% glutaraldehyde, 2% paraformaldehyde in 0.1 M potassium phosphate buffer pH7.4 overnight, dehydrated with a graded ethanol series from 25% to 100%. Ovaries were cut transversely in the middle with sharp blade after critical point drying and before platinum coating. Samples were scanned and imaged recorded with Hitachi S-3500N scanning electron microscope (Hitachi Ltd, Japan) under high vacuum. Ovary sizes were measured with the images using ImageJ software. Total yield of ripe and green fruit were recorded separately by the weight and number of the fruit according to previously established protocols [57]. Fruit ripening was recorded with hand pollinated first two flowers of each inflorescence. Six to ten fruits per plant that set well were evaluated. The dates were recorded as each fruit turned to orange (30%-60% surface color change to orange) and red (>90% surface color change to red). Fruit quality was measured as the total soluble solid content (degree of Brix). A quarter of each 14 representative ripe fruits were blended together per plant. The homogenized juice was filtered with Kimwipes and Brix was measured by a pocket refractometer (ATAGO CO., LTD, Tokyo, Japan). Seeds were extracted from fully ripe fruit and soaked in 12.5% HCl, rinsed and air dried in the laboratory for 6 days on mesh screens with paper towels, weighted and counted. Inflorescence number and flower number per inflorescence per plant were counted with greenhouse grown plants under 20-20-20 with Calcium supplement or 14-7-14 fertilizer condition. Developing inflorescences and aborted flowers were also included. Plant architecture was measure as plant height, node number, side shoot number and total side shoot length at 55 and 66 days after sowing (DAS) in the greenhouse and 87 and 108 DAS or 42 and 63 days after transplanting (DAT) in the field. Leaf structure were measured with the mature leaves at 8th, 9th, and 10th nodes counting from cotyledon. Leaf weight, rachis length, petiole length, intercalary leaflet number, secondary leaflet number, tertiary leaflet number and terminal leaflet size (width and length) were measured. To test source-sink relationship, two fruits per inflorescence of a total of 7 inflorescences were kept and fruit weight from these plants was compared with control plants with no fruit removal. All phenotypic evaluations were performed with *fw11*.*3* NILs under RG background with two replications except source-sink relationship experiment, each with 6 to 13 plants (S3 Table). Student *t*-tests were performed to compare each trait between *fw11*.*3*-D and *fw11*.*3*-WT NIL.

### CSR protein sequence analysis and phylogeny tree building

To identify conserved domains in the CSR protein, the Conserved Domain Database (CDD, https://www.ncbi.nlm.nih.gov/cdd/) [58] was used. The FAF domain (Pfam accession PF11250) was identified in 14 proteins in tomato genome protein sequence ITAG2.4 release (SGN, http://solgenomics.net/tools/blast/; Solyc01g009260.1.1, Solyc01g009270.1.1, Solyc01g079740.2.1, Solyc01g098570.2.1, Solyc06g008990.1.1, Solyc06g054310.1.1, Solyc06g073940.2.1, Solyc06g074270.1.1, Solyc06g084280.1.1, Solyc09g065140.1.1, Solyc10g018270.1.1, Solyc11g068530.1.1, and Solyc11g071940.1.1 (the latter corresponds to *CSR*). Thirteen of the tomato proteins were used in this study, except Solyc04g072650.1.1, which appeared to be an outlier in the protein phylogeny. To ensure the accuracy of protein sequences, all encoded proteins were confirmed using the predicted mRNA sequences with the ExPASy translation tool [59]. Additional motif searches were conducted using the full length protein sequence in MEME 4.10.0 [60] using the settings of 6 different motifs with 6–50 motif width. Three predicted proteins (Solyc06g073940.2.1, Solyc01g009260.1.1, and Solyc01g009270.1.1) featured the most similar motif patterns and highest similarity with CSR (*E*-value ≤ 1e-80), and therefore these were defined as paralogs. The FAF domain was found in 10 Arabidopsis proteins (TAIR, http://www.arabidopsis.org/wublast/index2.jsp) and seven of them were used in this study. Of these, only one was considered the closest paralogs of CSR because of high MEME motif similarities. Four FAF domain-containing Arabidopsis proteins corresponded to the FANTASTIC FOUR (FAF) clade.

Multiple sequence alignments of FAF by CLUSTALW2 [61] with default settings were performed and the results were exported to MEGA6 [62] for phylogenetic analysis. Neighbor-joining tree was constructed for 13 tomato and seven Arabidopsis FAF domain sequences with 1000 replicates for bootstrap validation. A FAF domain from *Selaginella moellendorffii* (Phytozome ID: 418746) was used as outgroup. Two proteins (Solyc01g079740.2.1 and Solyc06g054310.1.1) were closely related to FAF3 and FAF4 and were renamed SlFAF3/4a and SlFAF3/4b. Three proteins (Solyc06g084280.1.1, Solyc06g008990.1.1 and Solyc09g065140.1.1) were also closely related and presented the FAF1 and FAF2 subclade, and were renamed to SlFAF1/2a, SlFAF1/2b, and SlFAF1/2c, respectively.

To identify potential orthologs in other crop species, the full length protein sequence of CSR-WT and its three paralogs were used as a query using the SGN database (http://solgenomics.net/tools/blast/), Cucurbit Genomics Database (http://www.icugi.org/cgi-bin/ICuGI/tool/blast.cgi), Phytozome version 9.1 (http://www.phytozome.net/) and Genome Database for Rosaceae (GDR; http://www.rosaceae.org/) and paralogs were identified in potato, eggplant, pepper, cocoa, sesame, mimulus, watermelon, cucumber, grape, poplar, peach, and strawberry (*E*-value ≤ 1e-80). MEME analysis was conducted to identify the most likely orthologs in each species by selecting those with the most similar motif patterning proteins. Multiple sequence alignment and phylogeny tree construction were performed as the same method described above.

### Fruit tissue collection for expression analysis

Three to thirty fruit from 10 *fw11*.*3*-WT and 10 *fw11*.*3*-D NIL plants each were collected between 1:00 pm to 3:00 pm and dissected tissues were immediately frozen in liquid nitrogen. The three tissues dissected were pericarp, columella, and developing seeds with placenta at the following developmental stages: 4, 7, 10, 15, 25, 33 DPA and turning stage fruit. For 4 DPA fruit, the columella, placenta and developing seeds were collected together. Most samples consisted of four replicates, except 7 DPA and 25 DPA (three replicates), 33 DPA (one replicate) and turning stage (two replicates). The low number of replicates for the latter tissues was due to severe incidence of blossom-end rot in the greenhouse.

### RNA isolation, RNA-seq library preparation and sequencing

Hot borate RNA extraction method [63] was used for total RNA extraction from 25, 33 DPA and turning stage fruit. The TRIzol (Invitrogen, Carlsbad, CA) RNA extraction method was used for 4, 7, 10 and 15 DPA fruit following the manufacturer’s specifications. RNA quantity and quality were assessed using a Qubit 2.0 fluorometer RNA Assay Kit (Invitrogen Inc. USA) and an Agilent 2100 Bioanalyzer RNA 6000 Nano kit (Agilent, USA). Approximately 5μg total RNA was used to prepare strand-specific libraries of approximately 250bp fragments [64, 65]. Libraries were barcoded and 8 samples were pooled per lane on the flowcell. Fifty-one bp single-end reads were generated on the Illumina HiSeq2000 at Genomics Resources Core Facility at Weill Medical College (New York, NY).

### Read alignments and library quality checks

The pre-processing, read alignment and quantification of gene levels were performed using previously established protocols in the lab [64] with minor modifications. Briefly, ribosomal RNA-free reads were mapped to the *Solanum lycopersicum* reference genome (Build SL2.50) using tomato gene model annotation (ITAG2.4 release) to facilitate mapping reads across exon-exon junctions. The final expression data were shown as reads per kilobase of exon model per million mapped reads (RPKM). For the expression of selected genes at different developmental stages, the average RPKM were used. Summary statistics for each of the RNA-seq libraries are shown in S8 Table.

The correlations among samples were evaluated because the results would address reproducibility among samples (S9 Table). The columella tissue at the turning stage (TCol) showed low correlation between the two replicates (64% for *fw11*.*3*-WT and 85% for *fw11*.*3*-D, respectively). After ruling out the possibility of *fw11*.*3*-D or *fw11*.*3*-WT sample switch by evaluating the SNPs at *fw11*.*3*, we found that unlike TCol_rep1, TCol_rep2 was more correlated with other turning stage tissue types (pericarp and seed/placenta) than TCol_rep1. This suggested mixed up tissue samples for TCol_rep2. We therefore decided to discard TCol_rep2 and only use TCol_rep1 to represent turning stage columella tissue.

### Co-expression clustering and GO term enrichment

The gene expression data used for co-expression cluster analysis was the gene expression RPKM values of three fruit tissue types at seven developmental stages (six stages for columella tissue). The identification of co-expressed genes with *CSR* was done separately with *CSR*-D and *CSR*-WT alleles. The data was first pre-processed by Mfuzz [66] for normalization. The heatmap.2 function in R was then employed to generate a heatmap based on the normalized RPKM. Twelve clusters for *CSR*-D and 10 clusters for *CSR*-WT were identified visually based on the heatmap results. Fuzzy C-means clustering in Mfuzz was applied with cluster number as 12 and 10 (*CSR*-D and *CSR*-WT, respectively) and default settings. Soft clustering was chosen in “visualization” to generate clusters. Finally, the clustering results with the probabilities of each gene in each cluster were exported to Excel. Genes with the probabilities below 90% were removed from the clusters.

Arabidopsis ortholog genes were obtained for *CSR*-D and *CSR*-WT co-expressed overlapping genes by using BLASTP against TAIR10 amino acid sequence (*p*-value ≤ 7.00E-06). Cytoscape plug-in ClueGO (Version 2.3.2) [67] was used to perform the Gene Ontology (GO) analysis using the GO biological process available on November 17 2016. The ClueGO networks were set to ‘medium’ and their connectivity was based on a kappa score of 0.4. GO Term grouping was selected with an initial group size of 1 and group merging set at 50%. Two-sided hypergeometric tests were applied and *p*-value correction was carried out using the Bonferroni step-down method. GO terms with adjusted *p* ≤ 0.05 were considered as significant.

### Accession numbers

Sequence data from this article can be found in the EMBL/GenBank data libraries under accession number SRP017242, SRP089936, SRP089970.

### Data records

The raw FASTQ files for the RNA-seq libraries were deposited at NCBI Sequence Read Archive (SRA) with SRA study accession SRP089936. Gene expression data (RPKM) are available through a Tomato Functional Genomic Database (TFGD; http://ted.bti.cornell.edu/cgi-bin/TFGD/digital/home.cgi).

## Supporting information

S1 Fig Flow cytometry of tomato pericarp nuclei. The single nuclei gating (upper) and nuclei ploidy histogram (lower) is shown for each genotype. In the upper graphs, the P1 box was manually set prior to counting the single nuclei that showed different ploidy levels (non-red dots). Colors were automatically generated by the flow cytometry BD FACSDIVA^TM^ software, and correspond to P2 to P9 nuclei. Red color represent debris. P2 represents 2C nuclei and was difficult to discern among the debris. P3 to P10 represent polyploid nuclei ranging from 4C to 512C shown on the X-axis. The widths of each nuclei level were manually adjusted. The horizontal bars in the histogram showed the area used for the nuclei counts.
(PDF)

S2 Fig Expression of *CSR* in fruit tissues. (A) Expression of *CSR* in developing fruit tissues from anthesis to ripe fruit. (B) Expression of *CSR* in developing pericarp tissues after fruit set.
(PDF)

S3 Fig *CSR* co-expression analysis. (A) *fw11*.*3*-D NIL gene expression cluster analysis. *CSR*-D and its co-expressed genes are included in cluster 11. (B) *fw11*.*3*-WT NIL gene expresssion cluster analysis. *CSR*-WT and its co-expressed genes are included in cluster 1. Normalized RPKM were used. Horizontal axis represents the following tissues and stages: 7Col (1), 10Col, 15Col, 25Col (4), 33Col, TCol, 4Per (7), 7Per, 10Per, 15Per, 25Per (11), 33Per, TPer, 4S, 7SPl, 10SPl (16), 15SPl, 25SPl, 33SPl, TSPl. (Col: columella; Per: pericarp; SPl: seeds and placenta. Numbers in parenthesis are show in the figure as x-axis labels).
(PDF)

S4 Fig Pedrigree of tomato plants used in the study. (A) Pedigree of plants used for progeny test, fine-mapping and genetic transformation in Howard German background. PP: *fw11*.*3*-WT NIL. (B) Pedigree of plants used for fine-mapping and phenotypic evaluations in Rio Grande background. (C) Pedigree of LA1589 fruit weight NILs.
(PDF)

S1 Table Progeny test of the selected recombinants in the *fw11*.*3* region. (XLSX)

S2 Table Fruit weight data from CSR transgenic and non-transgenic lines. (XLSX)

S3 Table Phenotypic characterizations of the *fw11*.*3* NILs. (XLSX)

S4 Table *fw11*.*3* NIL ploidy level analyses in the pericarp and columella. (XLSX)

S5 Table Expression of *CSR* and *CSR*-*like* genes. (XLSX)

S6 Table Marker and primer information. (XLSX)

S7 Table Association mapping core collection and genotypes for *fw11*.*3* region. (XLSX)

S8 Table Summary statistics of the RNA-seq libraries. (XLSX)

S9 Table RNA-seq samples correlation among replicates. (XLSX)

S1 Dataset *CSR* coexpressed gene list with Arabidopsis annotations. (XLSX)

S2 Dataset CSR GO term enriched gene list. (XLSX)
