# Supplementary figures and images for: Fruit weight is controlled by Cell Size Regulator encoding a novel protein that is expressed in maturing tomato fruits
Source: PLoS Genet. 2017 Aug 17;13(8):e1006930. doi: 10.1371/journal.pgen.1006930 (PMC5560543; doi:10.1371/journal.pgen.1006930)

Specimen 1-3-3P

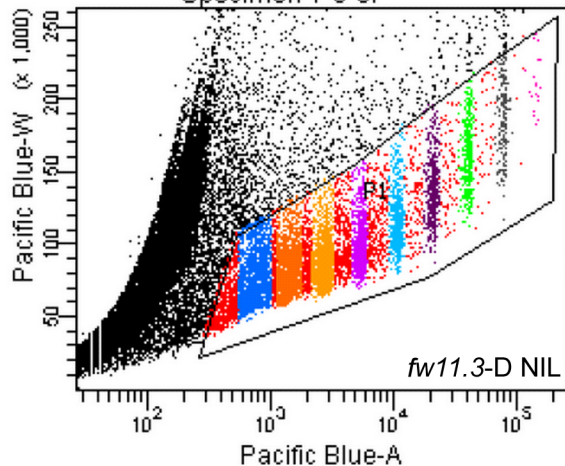

Specimen 1-44-3P

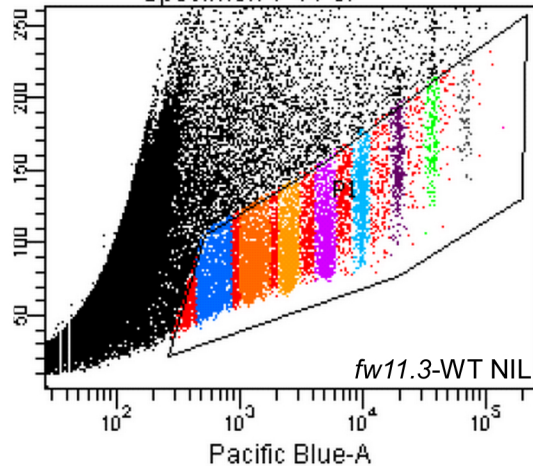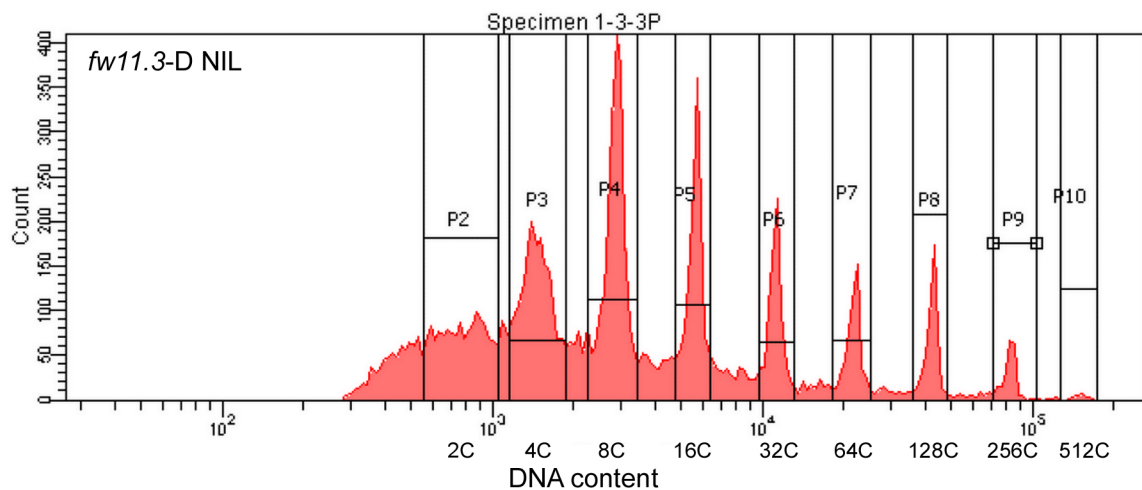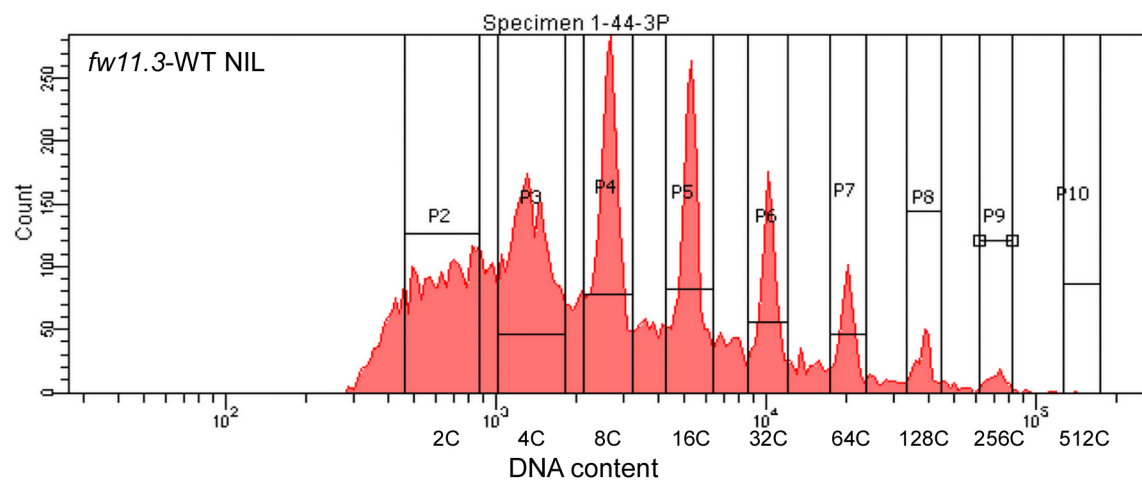

Supplement: S1 Fig — The single nuclei gating (upper) and nuclei ploidy histogram (lower) is shown for each genotype. In the upper graphs, the P1 box was manually set prior to counting the single nuclei that showed different ploidy levels (non-red dots). Colors were automatically generated by the flow cytometry BD FACSDIVATM software, and correspond to P2 to P9 nuclei. Red color represent debris. P2 represents 2C nuclei and was difficult to discern among the debris. P3 to P10 represent polyploid nuclei ranging from 4C to 512C shown on the X-axis. The widths of each nuclei level were manually adjusted. The horizontal bars in the histogram showed the area used for the nuclei counts. (PDF) [file pgen.1006930.s001.pdf]

**S4A Fig**

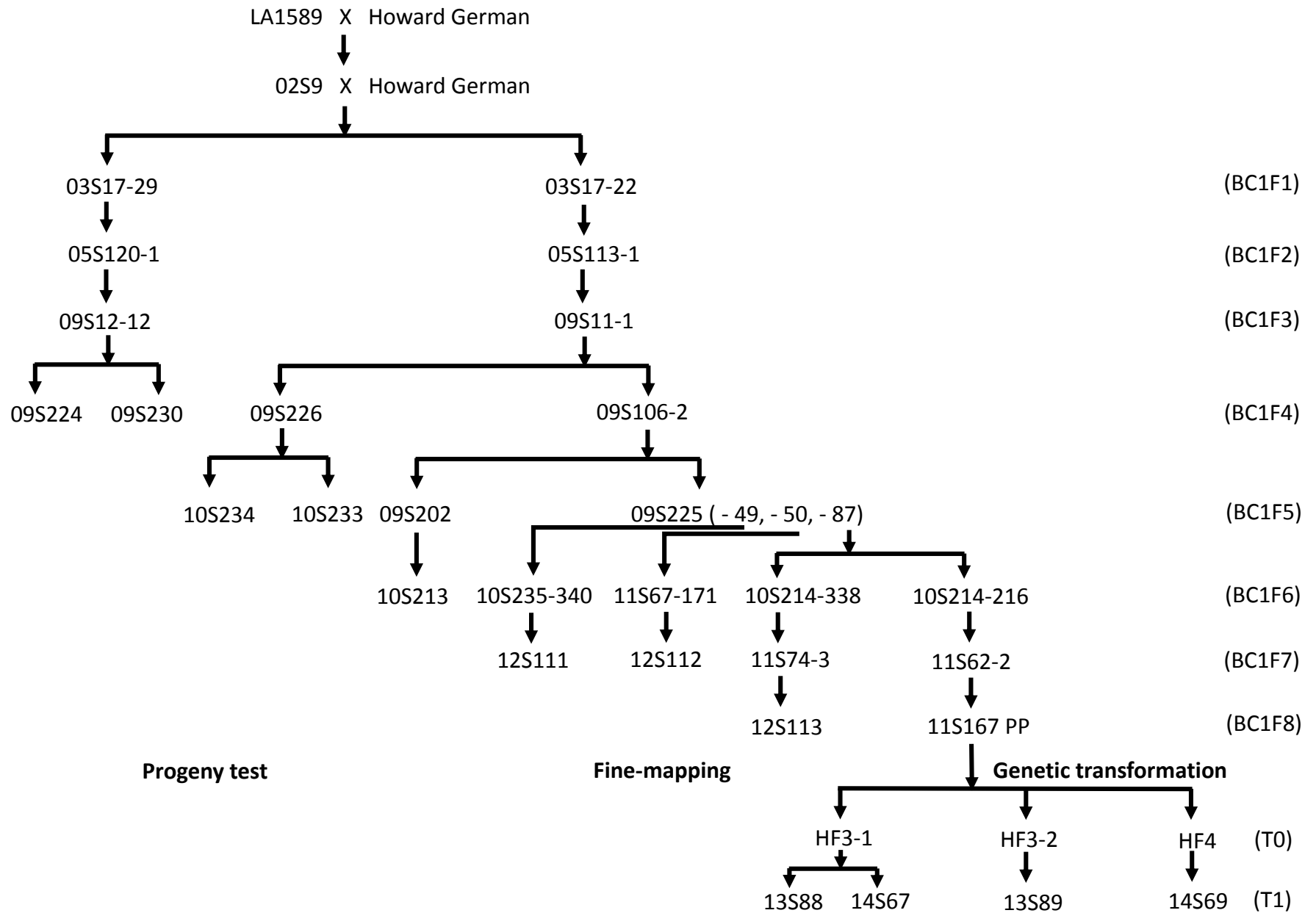

**S4B Fig**

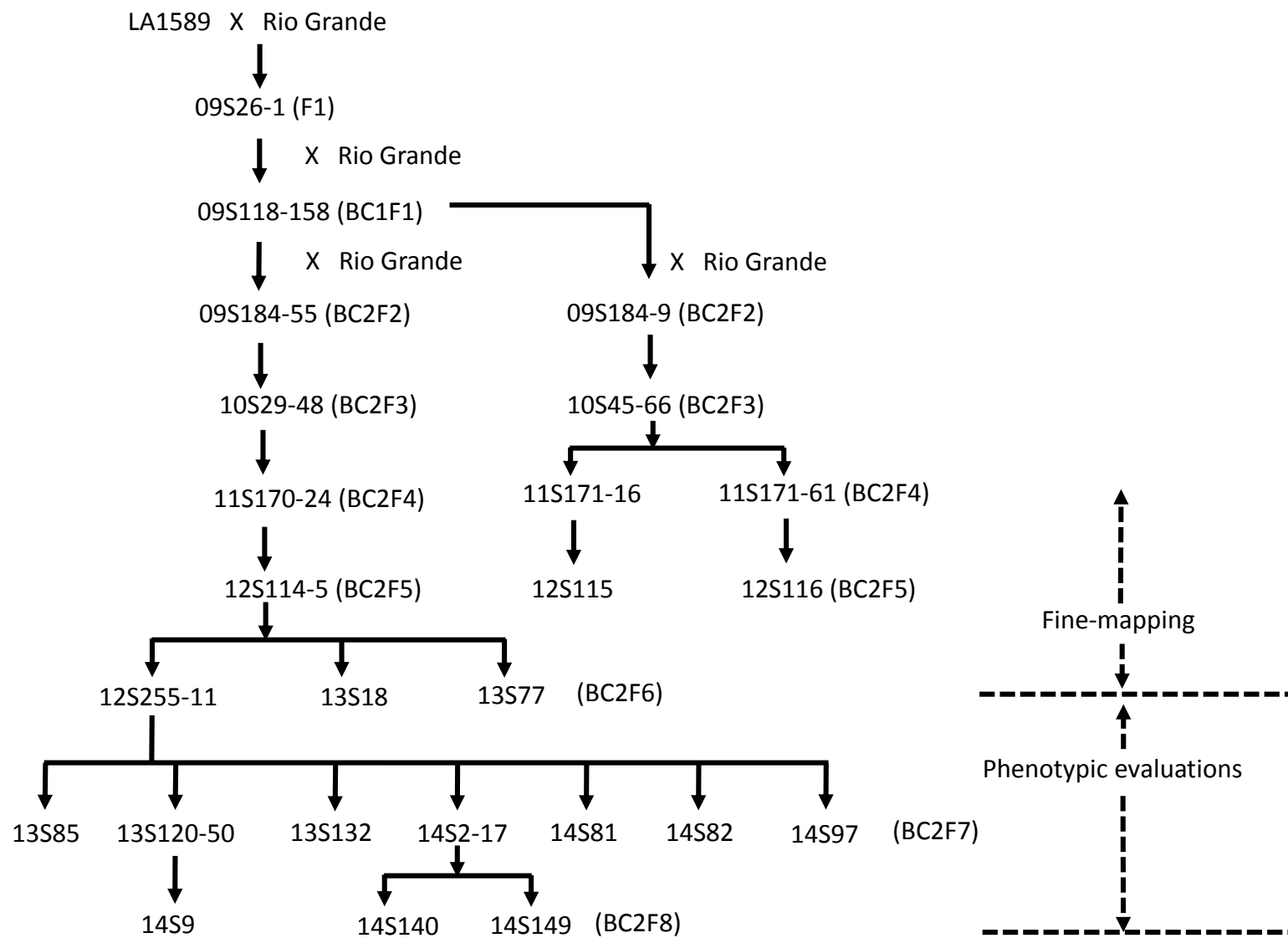

S4C Fig

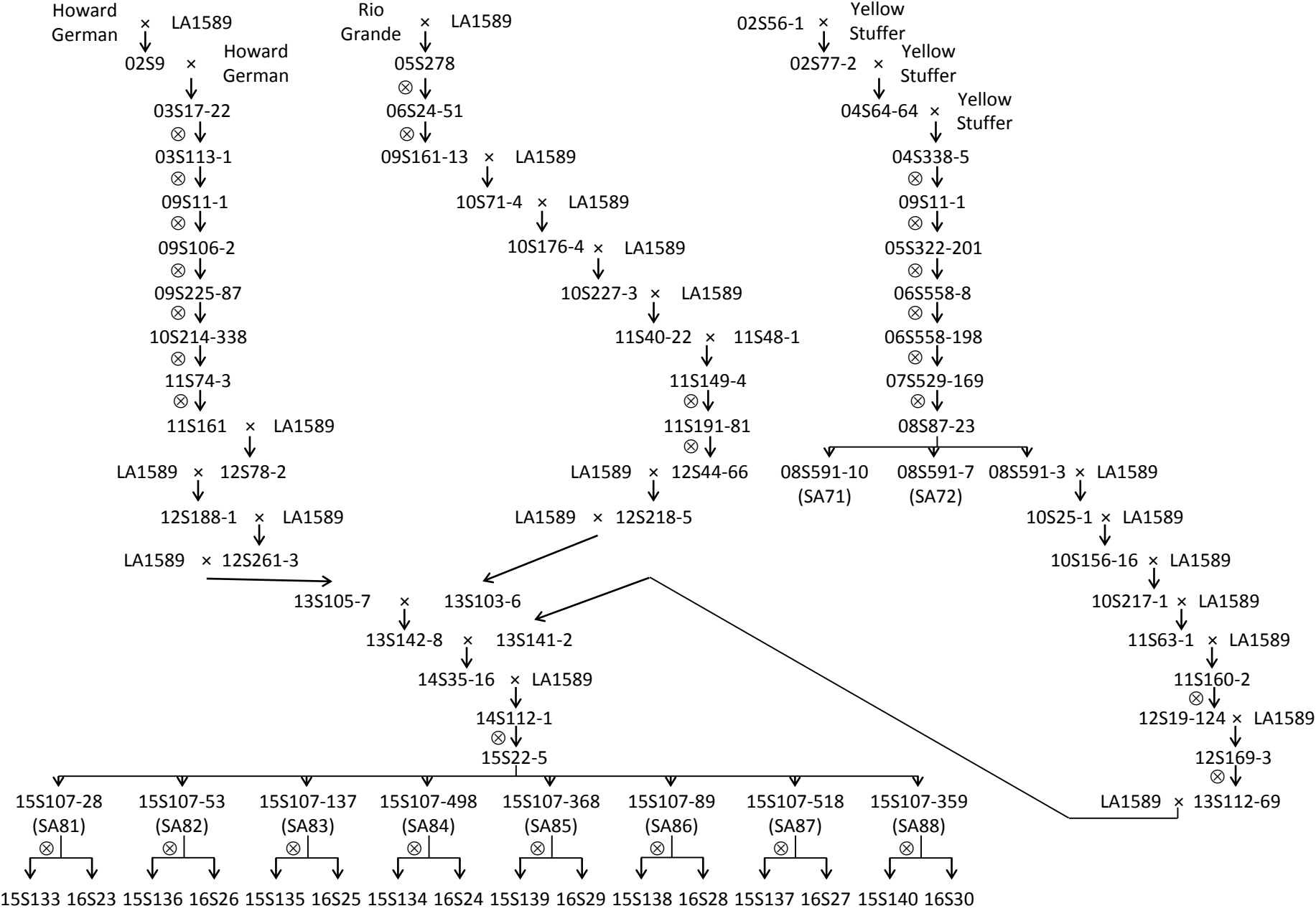

Supplement: S4 Fig — (A) Pedigree of plants used for progeny test, fine-mapping and genetic transformation in Howard German background. PP: fw11.3-WT NIL. (B) Pedigree of plants used for fine-mapping and phenotypic evaluations in Rio Grande background. (C) Pedigree of LA1589 fruit weight NILs. (PDF) [file pgen.1006930.s004.pdf]
