# Supplementary material for: Fruit weight is controlled by Cell Size Regulator encoding a novel protein that is expressed in maturing tomato fruits
Source: PLoS Genet. 2017 Aug 17;13(8):e1006930. doi: 10.1371/journal.pgen.1006930 (PMC5560543; doi:10.1371/journal.pgen.1006930)
Supplement: S2 Fig — (A) Expression of CSR in developing fruit tissues from anthesis to ripe fruit. (B) Expression of CSR in developing pericarp tissues after fruit set. (PDF) [file pgen.1006930.s002.pdf]

S2A Fig. Expression of *CSR* in developing fruit tissues

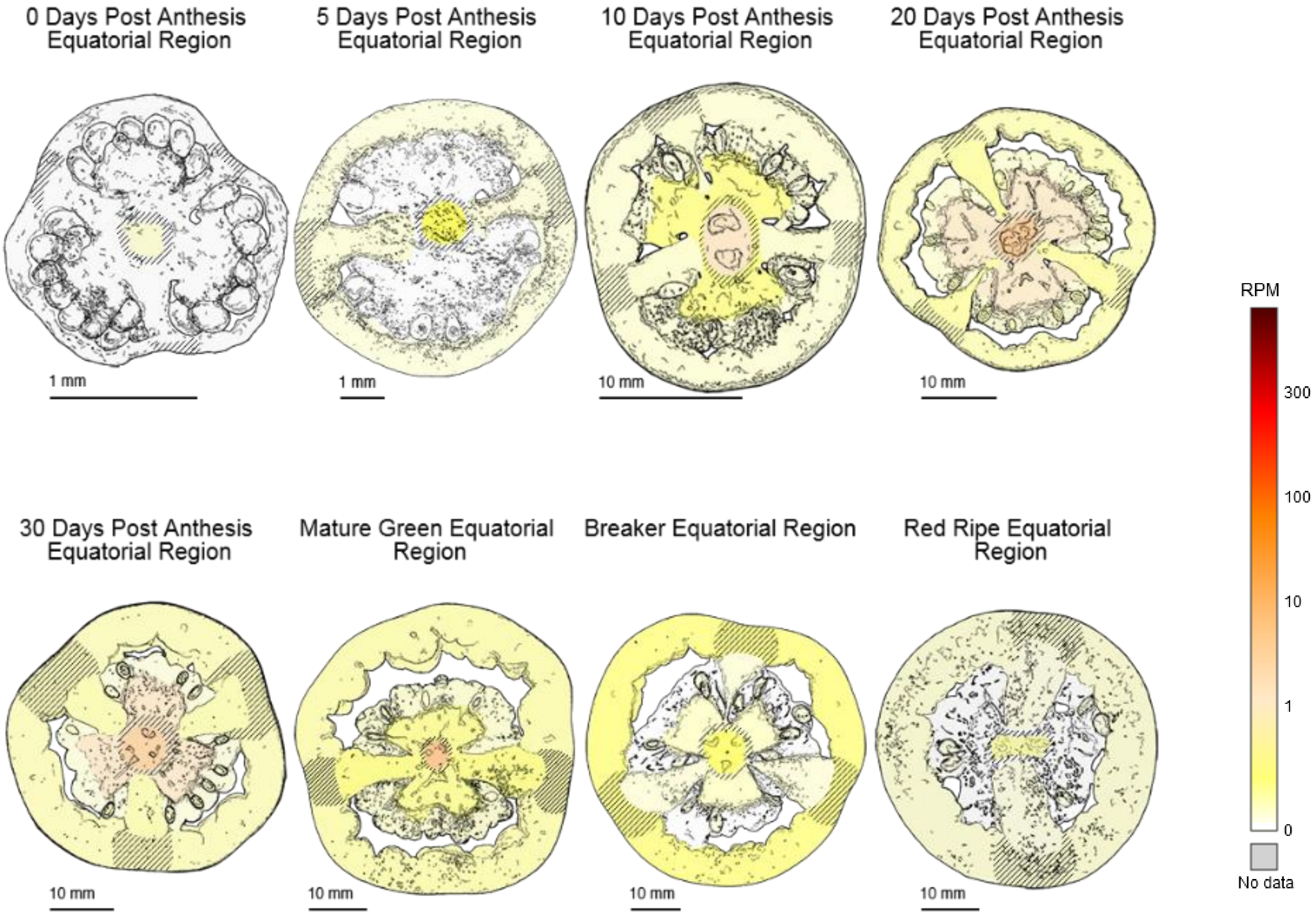

http://tea.solgenomics.net

S2B Figure. Expression of *CSR* in developing pericarp tissues

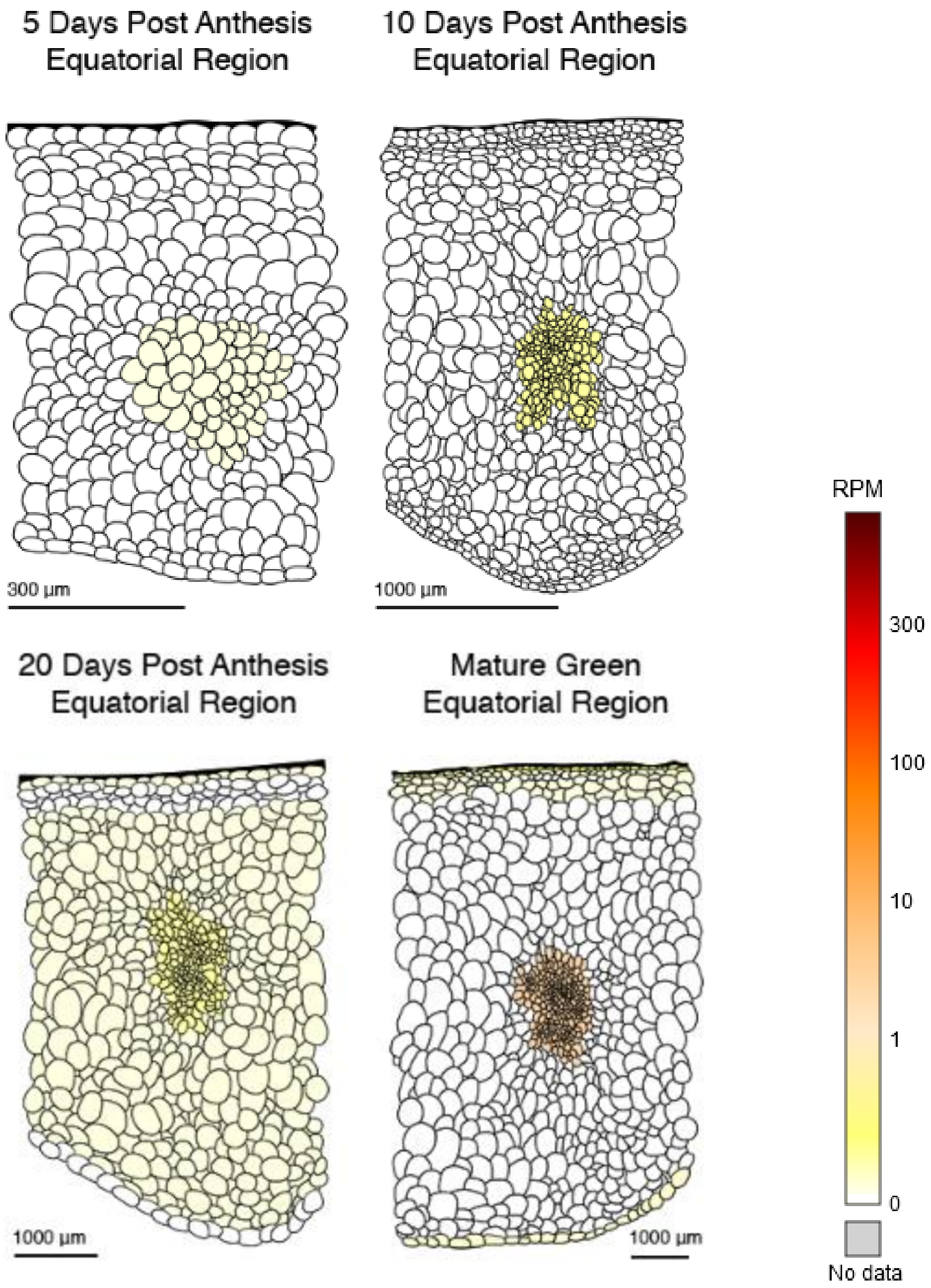

http://tea.solgenomics.net
