# Supplementary material for: Fruit weight is controlled by Cell Size Regulator encoding a novel protein that is expressed in maturing tomato fruits
Source: PLoS Genet. 2017 Aug 17;13(8):e1006930. doi: 10.1371/journal.pgen.1006930 (PMC5560543; doi:10.1371/journal.pgen.1006930)
Supplement: S3 Fig — (A) fw11.3-D NIL gene expression cluster analysis. CSR-D and its co-expressed genes are included in cluster 11. (B) fw11.3-WT NIL gene expresssion cluster analysis. CSR-WT and its co-expressed genes are included in cluster 1. Normalized RPKM were used. Horizontal axis represents the following tissues and stages: 7Col (1), 10Col, 15Col, 25Col (4), 33Col, TCol, 4Per (7), 7Per, 10Per, 15Per, 25Per (11), 33Per, TPer, 4S, 7SPl, 10SPl (16), 15SPl, 25SPl, 33SPl, TSPl. (Col: columella; Per: pericarp; SPl: seeds and placenta. Numbers in parenthesis are show in the figure as x-axis labels). (PDF) [file pgen.1006930.s003.pdf]

S3A Fig

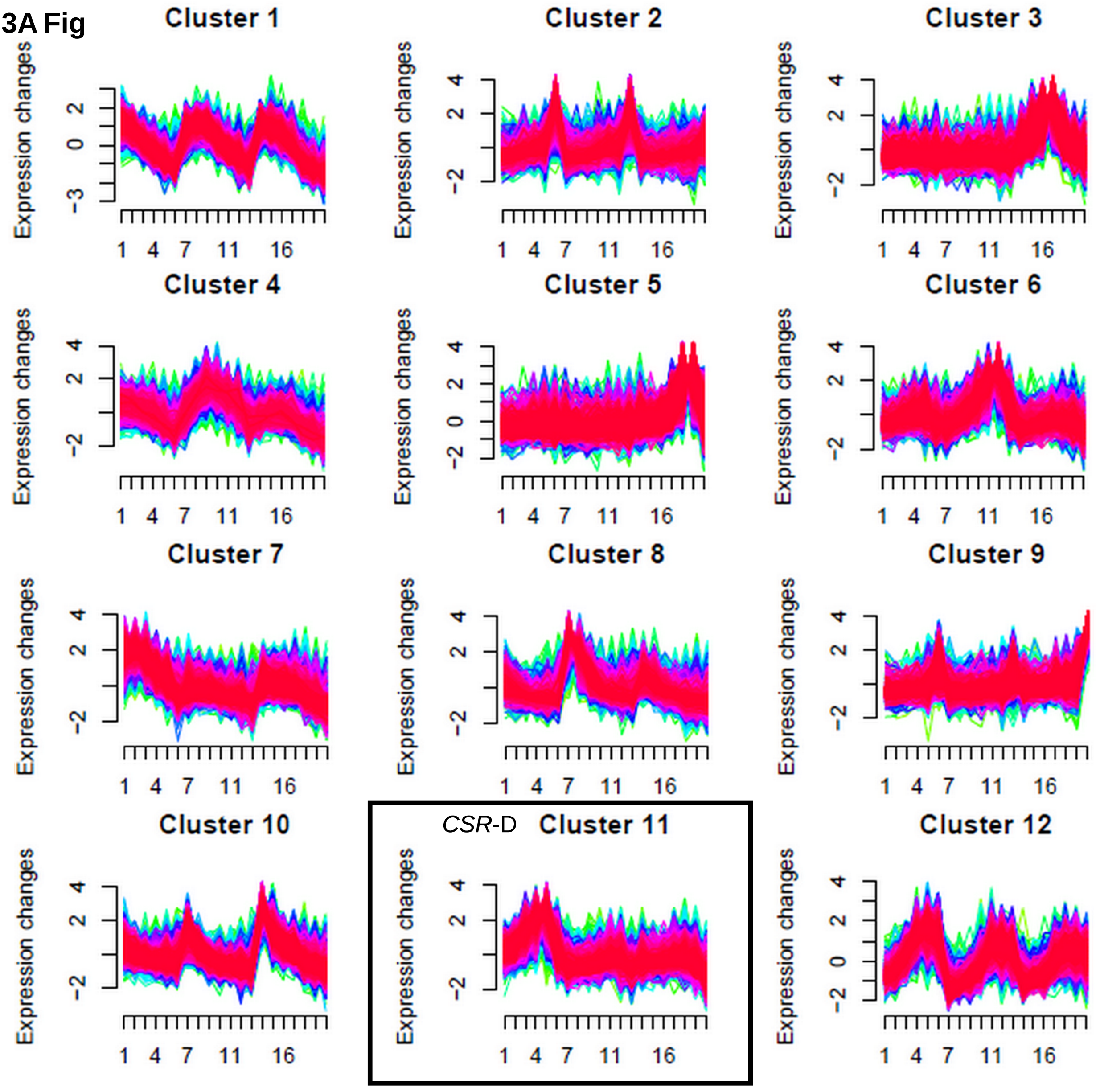
Cluster 1
Cluster 2
Cluster 3
Cluster 4
Cluster 5
Cluster 6
Cluster 7
Cluster 8
Cluster 9
Cluster 10
CSR-D Cluster 11
Cluster 12
Expression changes
1 4 7 11 16

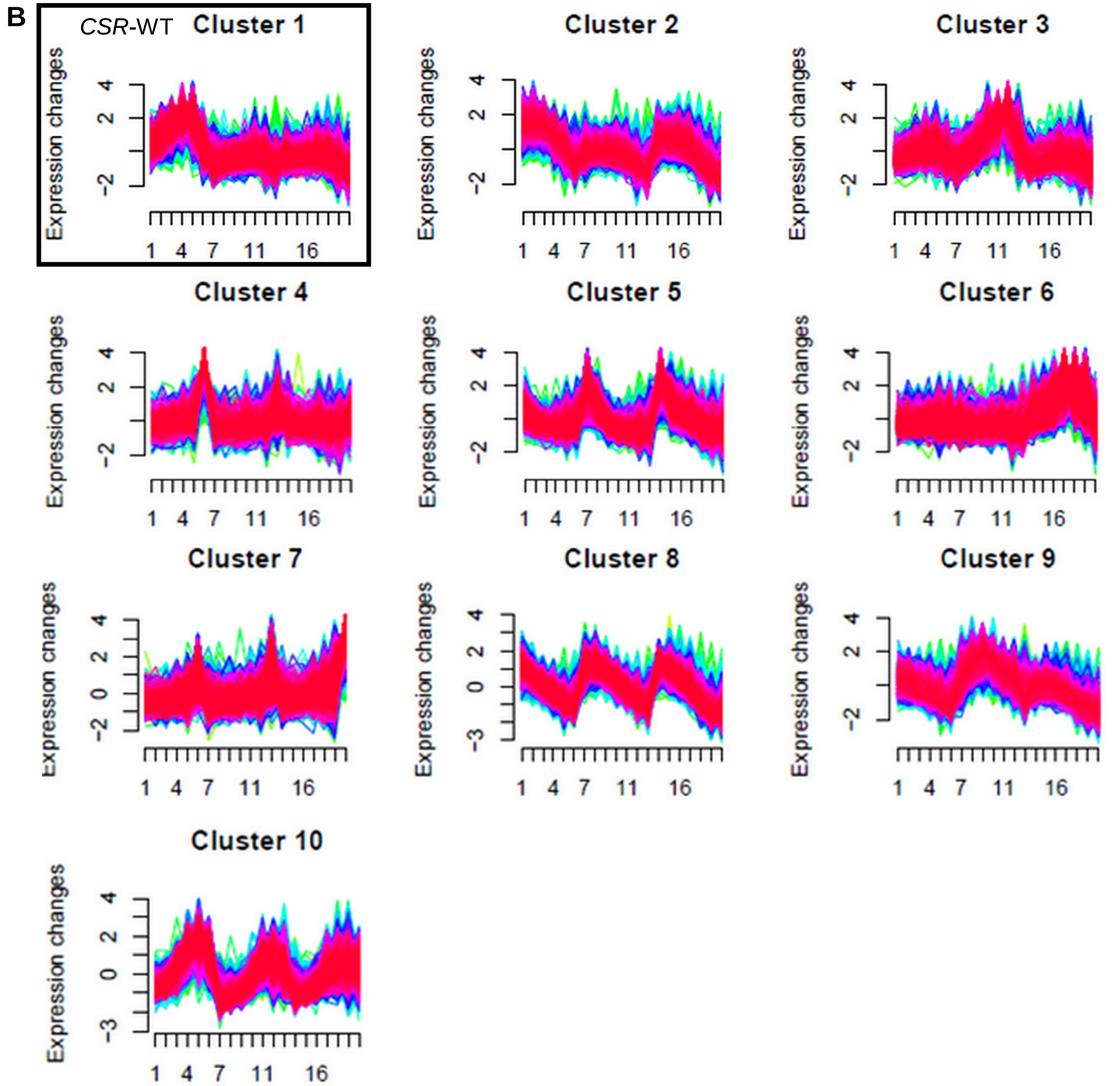
B
CSR-WT
Cluster 1
Cluster 2
Cluster 3
Cluster 4
Cluster 5
Cluster 6
Cluster 7
Cluster 8
Cluster 9
Cluster 10
Expression changes
1 4 7 11 16
